# Variation in global trauma care: a survey of 187 hospitals across 51 countries

**DOI:** 10.1136/bmjgh-2025-021784

**Published:** 2025-11-09

**Authors:** Thomas Edmiston, Michael F Bath, Eder Caceres, Carlos M Nuño-Guzmán, Daniel Umugisha Baderhabusha, Monty Khajanchi, Joachim Amoako, Katharina Kohler, Abdullahi Said Hashi, Luca Carenzo, Zhongheng Zhang, Max Marsden, Raoof Saleh, Charlotte C Hammer, Laura Hobbs, Brandon G Smith, Peter Hutchinson, Thomas Weiser, Zane B Perkins, Timothy Craig Hardcastle, Tom Bashford, on behalf of the GOAL-Trauma Collaborative, Aiman Aamir

**Affiliations:** 1 International Health Systems Group, Department of Engineering, University of Cambridge, Cambridge, UK; 2 Critical Care Department, Clínica Universidad de La Sabana, Chía, Colombia; 3 Hospital Civil de Guadalajara Unidad Hospitalaria Fray Antonio Alcalde, Guadalajara, Mexico; 4 Centro Universitario de Ciencias de la Salud, Universidad de Guadalajara, Guadalajara, Mexico; 5 Hôpital de Kyeshero, Goma, Congo (the Democratic Republic of the); 6 Department of Surgery, King Edward Memorial Hospital and Seth Gordhandas Sunderdas Medical College, Mumbai, India; 7 University of Ghana Medical School, Accra, Ghana; 8 Department of Surgery, Korle Bu Teaching Hospital, Accra, Ghana; 9 Department of Anaesthesia, Cambridge University Hospitals NHS Foundation Trust, Cambridge, UK; 10 Department of Anesthesiology and Reanimation, Mogadishu Somali-Türkiye Recep Tayyip Erdoğan Training and Research Hospital, Mogadishu, Somalia; 11 IRCCS Istituto Clinico Humanitas, Milan, Italy; 12 Department of Emergency Medicine, Zhejiang University School of Medicine Sir Run Run Shaw Hospital, Hangzhou, Zhejiang, China; 13 School of Medicine, Shaoxing University, Shaoxing, Zhejiang, China; 14 Major Trauma Service, The Royal London Hospital, London, UK; 15 Médicins Sans Frontières Medical Unit, Berlin, Germany; 16 Centre for the Study of Existential Risk, Institute for Technology and Humanity, University of Cambridge, Cambridge, UK; 17 NIHR Global Health Research Group on Acquired Brain and Spine Injury, Cambridge, UK; 18 Department of Clinical Neurosciences, University of Cambridge, Cambridge, UK; 19 Department of Surgery, Stanford University School of Medicine, Stanford, California, USA; 20 Centre for Trauma Sciences, Blizard Institute, Queen Mary University of London, London, UK; 21 Trauma and Burns Unit, Inkosi Albert Luthuli Central Hospital, Inkosi Albert Luthuli Central Hospital, Durban, South Africa; 22 Department of Surgical Sciences, Nelson R Mandela School of Clinical Medicine (NRMSM), University of KwaZulu-Natal, Durban, South Africa

**Keywords:** Injury, Health policy, Health systems, Health systems evaluation, Surgery

## Abstract

**Background:**

Trauma is a heterogeneous disease entity, with high rates of mortality and morbidity observed globally. While the health systems in which trauma patients are cared for worldwide have been previously assessed to varying degrees, many of the system processes that shape trauma patient outcomes remain unknown.

**Methods:**

We conducted a survey of 187 hospitals across 51 countries, through the GOAL-Trauma collaborative network. We explored prehospital, intrahospital and rehabilitation phases of trauma care. Data were compared across Human Development Index (HDI) tertiles, with thematic analyses performed to identify similarities and variation across settings.

**Findings:**

Hospital-based care appeared to develop preferentially out of the three phases of trauma care, with challenges from the middle HDI tertile being more analogous to the upper HDI tertile than the lower HDI tertile. A lack of emergency medical services, limited patient finances and a lack of health literacy were common causes of prehospital delay in the lower and middle HDI tertiles. Surgeons and anaesthetists working in lower and middle HDI tertiles perform approximately 3-fold and 10-fold more operations, respectively, compared with upper HDI tertile counterparts. Across all HDI tertiles, infection was reported as the most common cause of postoperative morbidity.

**Interpretation:**

A wide range of resources and processes exist globally in trauma care. Our findings suggest that with increasing resource availability, in-hospital care of trauma patients develops preferentially over prehospital or rehabilitation services. There is a clear need to coordinate available resources across all phases of care in order to improve outcomes among trauma patients.

**WHAT IS ALREADY KNOWN ON THIS TOPIC**

- The majority of trauma patients worldwide receive care outside of a formalised trauma system, with their healthcare shaped through more organic processes, the procedures and processes of which are poorly understood.

**WHAT THIS STUDY ADDS**

- There is significant global variation across all phases of trauma care.
- Lower resource settings appear to experience more financial-related and procurement-related issues, while higher resource settings appear to experience more systems-level and process-level issues.
- Hospital-based care appears to develop preferentially out of all the phases of trauma care.

**HOW THIS STUDY MIGHT AFFECT RESEARCH, PRACTICE OR POLICY**

- While there are clear targets for capacity building in trauma care from across the patient pathway, given the breadth of issues highlighted, use of a systems approach is essential if the overall standard of global trauma care is to be improved.

## Introduction

 Traumatic injuries result in 10% of all disability-adjusted life years globally^1^ and cause over 5 million deaths per year.^2^ Despite the disproportionately high number of trauma-related casualties in low- and middle-income countries (LMICs),^3^ it has been estimated that LMICs perform trauma surgery at a rate that is just 6% of the Lancet Commission on Global Surgery’s benchmark country.^4^ This is further compounded by a well-documented dearth of healthcare professionals (including surgeons) in LMICs.^5^ As a result, the need to improve trauma care and strengthen trauma systems globally has been identified as a priority by the World Health Assembly^6^—by improving trauma systems, medically preventable deaths can be reduced by up to 50%.^7^

However, development in global trauma care provision requires improvements to be made across the entire patient pathway. Comprehensive trauma care requires a complex system of intersecting processes and behaviours, all linking across co-existing healthcare services and regional infrastructure.^8^ A lack of emergency medical services, financial barriers and a lack of hospital infrastructure have previously been cited as individual factors that can limit a trauma service.^59^,^12^ However, when viewed through a wider lens, much less is known how their interactions impact overall trauma care at a systems level. Improvement to an individual aspect of trauma care is unlikely to improve overall patient outcomes in isolation, and a more holistic system-level view is often warranted.^8^

Despite the enormous global burden of trauma and its resultant economic impact,^13 14^ the care of trauma patients in LMICs remains an under-researched field of global health. As the vast majority of trauma patients worldwide receive care outside of a formal trauma system,^15^ their healthcare journey is shaped much more through organic processes, from which emerge even more complex patterns of care. Understanding these patterns and their inherent limitations is key to improving patient outcomes following trauma, yet there is still a relative paucity of published prospective data on this topic, especially in less-resourced settings.^16^ Evaluating such a complex system can be a challenging task, as all component parts and interactions must be assessed, not just individual patient pathways.

By surveying collaborators within an established global trauma research network, this study aimed to investigate the current characteristics, functions and limitations of hospitals worldwide that provide trauma care, across prehospital, intrahospital and rehabilitation phases of care.

## Methods

### Study design and setting

The GOAL-Trauma study was a prospective, international, multicentre, observational study, conducted between April 2024 and December 2024.^17^ The study received ethical approval from the Cambridge Psychology Research Ethics Committee (PRE.2023.119) and was registered to ClinicalTrials.gov (NCT06180668), with the study sponsored by the University of Cambridge (Cambridge, UK).

The study methodology has been previously described extensively.^18^ In brief, recruitment of hospitals was through open invitation, with potential collaborators contacted through a purposive snowballing technique, using pre-existing research networks, personnel contacts and social media. The study recruited 187 centres across 51 countries, representing each of the six inhabited continents and all strata in the World Bank country classification by income level.^19^ Each recruiting centre appointed a lead investigator (termed the ‘local lead’), who was invited to complete an online structured survey regarding trauma care at their institution.

The survey was distributed using a secure web-based system, REDCap cloud,^20 21^ and survey data were stored securely on the University of Cambridge password-protected servers, with no personally identifiable information involved. Only one response was permitted per hospital. The full survey can be found in the online supplemental material.

### Study outcomes

The survey was designed based on previous international multicentre collaborative research studies^22^ and core themes identified from global trauma guidelines.^23 24^ The survey was further refined through expert consensus, involving clinicians and academics across a range of specialties, including prehospital medicine, surgery, anaesthesia and intensive care, with experience across a range of income settings.

Study questions were categorised into four groups: hospital characteristics and provision; prehospital phase of care; preoperative and intraoperative phase of care; postoperative and rehabilitation phase of care. The prehospital phase of care was defined as being from the time of injury to the arrival at the treating centre, the preoperative and intraoperative phase of care as from arrival at the treating centre until the end of the initial treatment or index operation, and the postoperative and rehabilitation phase as the time after the initial treatment or index operation until recovery. Select aspects of the survey also included free text responses.

### Data analysis

Each hospital was stratified based on its national Human Development Index (HDI),^25^ with the HDI categorised into lower tertile, middle tertile and upper tertile. Data were summarised using mean and SD, median and IQR or number and percentage, where appropriate. Differences between HDI tertiles were assessed with ANOVA or χ² test where appropriate, with a p value <0.05 taken as the threshold of statistical significance.

Respondents were asked to report the number of trauma patients presenting, admitted and operated on, as well as the number of surgeons and anaesthetists. These numbers were screened for feasibility, and where inconsistencies were identified, local leads were contacted to clarify their responses; where no response was received, the data for these specific responses were excluded from analysis.

Inductive qualitative content analysis (QCA)^26^ was performed on the free text responses to distill recurring themes raised by the respondents. Ontologically, the QCA adopted a relativist position,^27^ recognising the existence of multiple and co-existing realities, whereby each participant could experience and describe their world differently.

### Patient and public involvement

A patient and public involvement (PPI) discussion group was convened for the study, with individuals who had specific lived experiences relevant to the project. Throughout the protocol development stage, feedback was obtained from the PPI group that directly influenced aspects of methodology.

## Results

### Hospital characteristics and provision

Responses were received from every participating hospital in the GOAL-Trauma study, with 54 hospitals from 15 lower HDI tertile countries, 50 hospitals from 16 middle HDI tertile countries and 83 hospitals from 20 upper HDI tertile countries (online supplemental material). There was a predominance towards urban hospitals (173 of 187 respondents, 92.5%), government-funded hospitals (162 of 187 respondents, 86.6%) and tertiary centres (139 of 173 respondents, 74.3%) in those surveyed. Half of the hospitals reported a population served of over one million (94 of 187 respondents, 50.3%).

When asked to rank which phase of trauma care management should be improved for the greatest impact on trauma outcomes, there were significant differences across tertiles (p<0.001); those in the lower HDI tertile preferentially reported prehospital care (29 of 54 respondents, 53.7%), respondents in the middle HDI tertile stated in-hospital care (24 of 50 respondents, 48%) and those in the upper HDI tertile stated rehabilitation (36 of 83 respondents, 43.4%).

**Figure 1:**
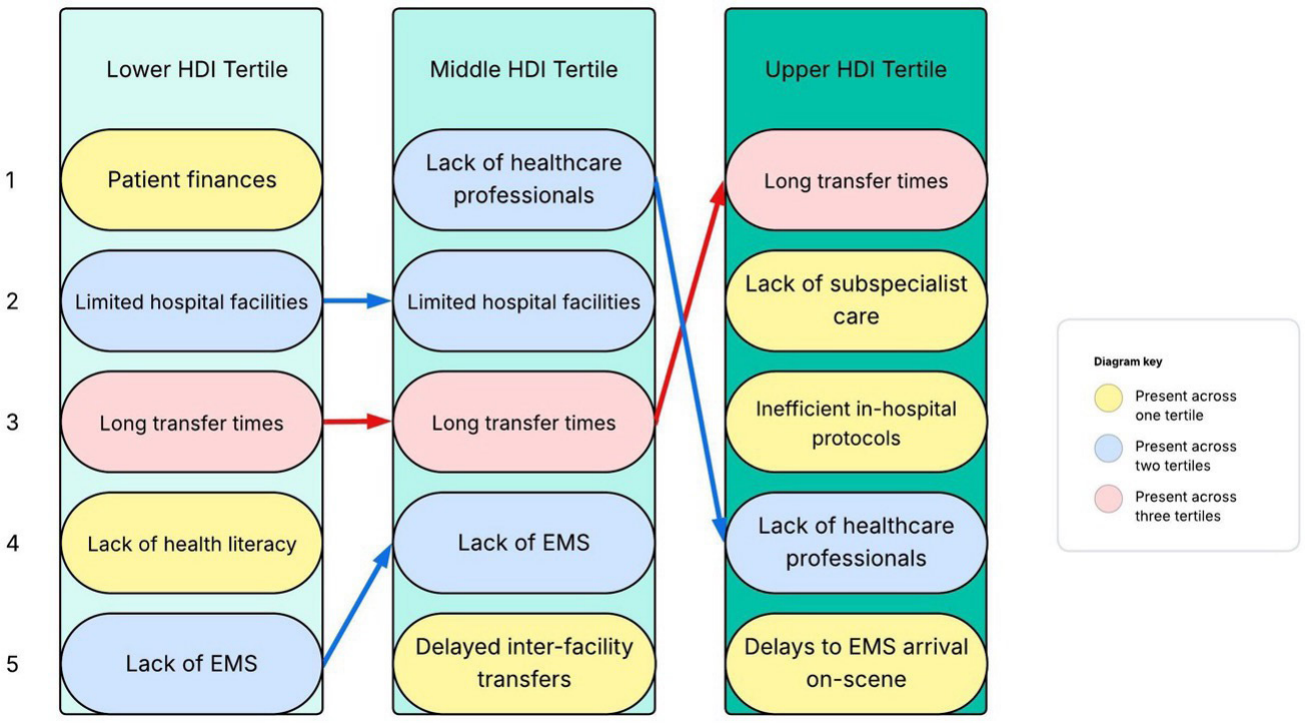
The most common barriers reported by respondents in accessing trauma care, numbered 1 to 5, stratified by HDI tertile. EMS, emergency medical services; HDI, Human Development Index.

When asked about the main limitations in accessing trauma care, half of the barriers mentioned across all tertiles occurred in the prehospital environment (figure 1). Long transfer times were reported across all the HDI tertiles as a key barrier. Similarities in the barriers reported were observed between lower and middle HDI tertile respondents, respectively, with three of the five most common barriers cited across both.

### Prehospital phase of care

**Figure 2:**
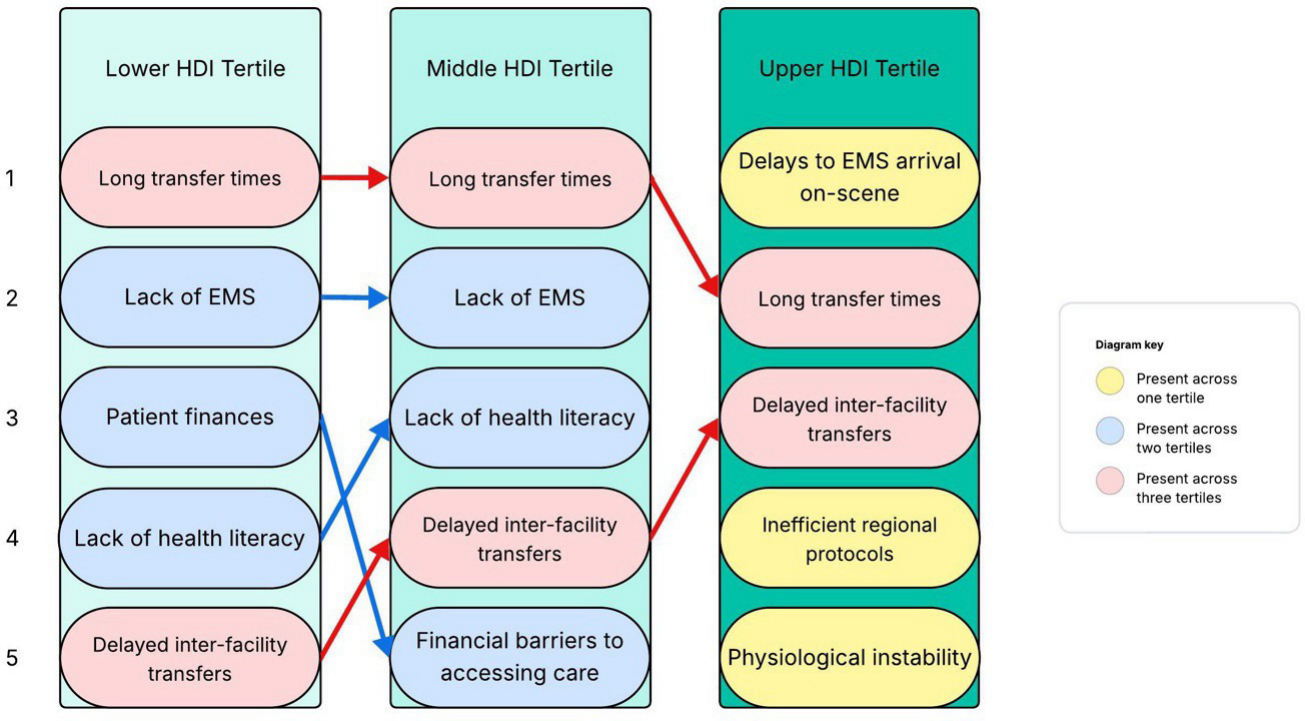
The most common reasons reported by respondents for delays in patients arriving to hospital following their initial injury, numbered 1 to 5, stratified by HDI tertile. EMS, emergency medical serviEmergency Medical Services; HDI, Human Development Index.

When asked for the most common reasons for delays in patients arriving to hospital following initial injury, prehospital transport and prehospital care were cited by respondents as common issues across all HDI tertiles (figure 2). While similar barriers were reported by both the lower HDI tertile and middle HDI tertile respondents, regional trauma system care and patient instability were issues reported uniquely by the respondents in the upper HDI tertile.

There was significant variation across HDI tertiles in the presence of a trauma team to assess seriously injured patients on arrival to hospital (χ² for trend, p<0.001), with the trauma team present ‘all of the time’ in 77.1% (64 of 83 respondents) of upper HDI tertile centres, compared with 60.0% (30 of 50 respondents) of middle HDI tertile centres and 37.0% (20 of 54 respondents) of lower HDI tertile centres. Similar trends were observed regarding the presence of a doctor with formal training in providing trauma care (p<0.001), with 66.3% (55 of 83 respondents) in the upper HDI tertile stating this would occur for the majority of cases, compared with 40.0% (20 of 50 respondents) in the middle tertile and 31.5% (17 of 54 respondents) in the lower HDI tertile.

### Preoperative and intraoperative phase of care

The availability of several key services was significantly higher in the upper HDI tertile compared with the middle and lower tertiles. Specifically, CT imaging services (χ² for trend, p<0.001), pathology services (p<0.001) and blood transfusion services (p=0.009) were all more available in the upper HDI tertile, with a similar pattern also evident for laparoscopic surgery (p<0.001) and interventional radiology (p<0.001).

**Figure 3:**
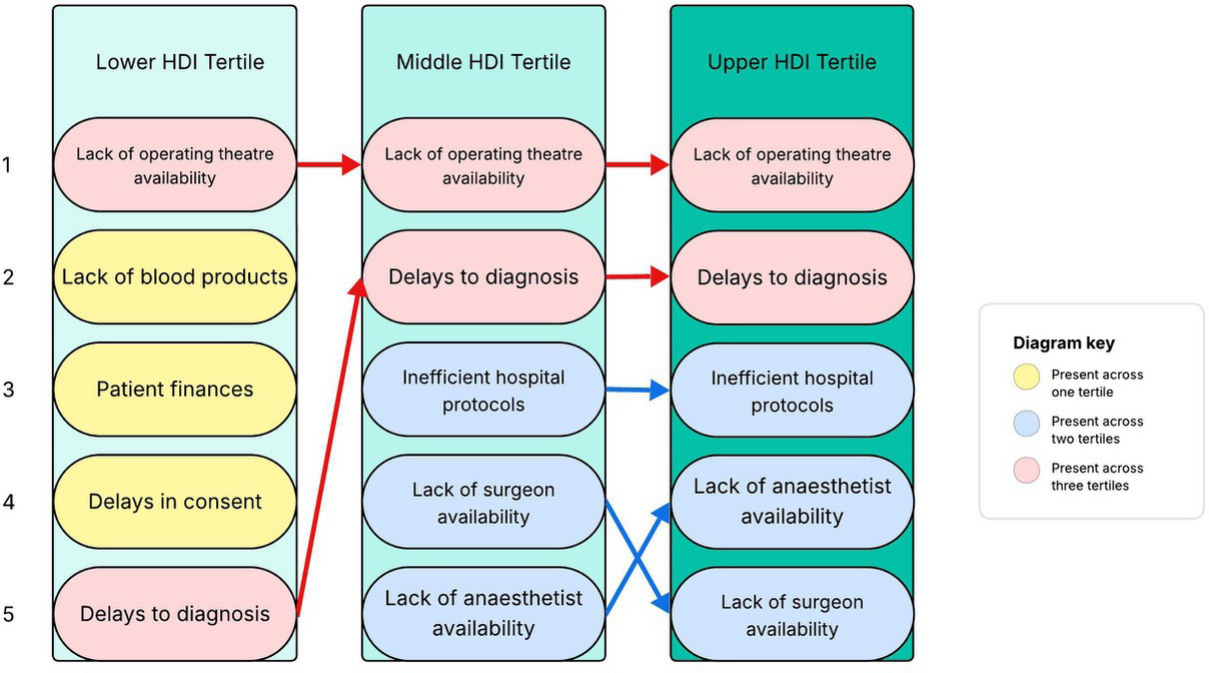
The most common reasons reported by respondents for delays in getting patients to the operating theatre for a trauma laparotomy, numbered 1 to 5, stratified by HDI tertile. EMS, emergency medical serviEmergency Medical Services; HDI, Human Development Index.

The most cited factor delaying transfer to the operating theatre for a trauma laparotomy was the lack of operating theatre availability (figure 3). Indeed, respondents from the middle HDI tertile and upper HDI tertile described the same five most common delays across these settings, while the lack of blood product availability and patient finances were more commonly cited by the lower HDI tertile respondents. A proportion of centres reported no delays in the transfer of a patient requiring a trauma laparotomy to theatre, with an increasing proportion from the lower HDI tertile respondents (5 of 54 centres, 9.3%) through to upper HDI tertile respondents (19 of 83 centres, 22.9%; χ² for trend p=0.027).

**Figure 4:**
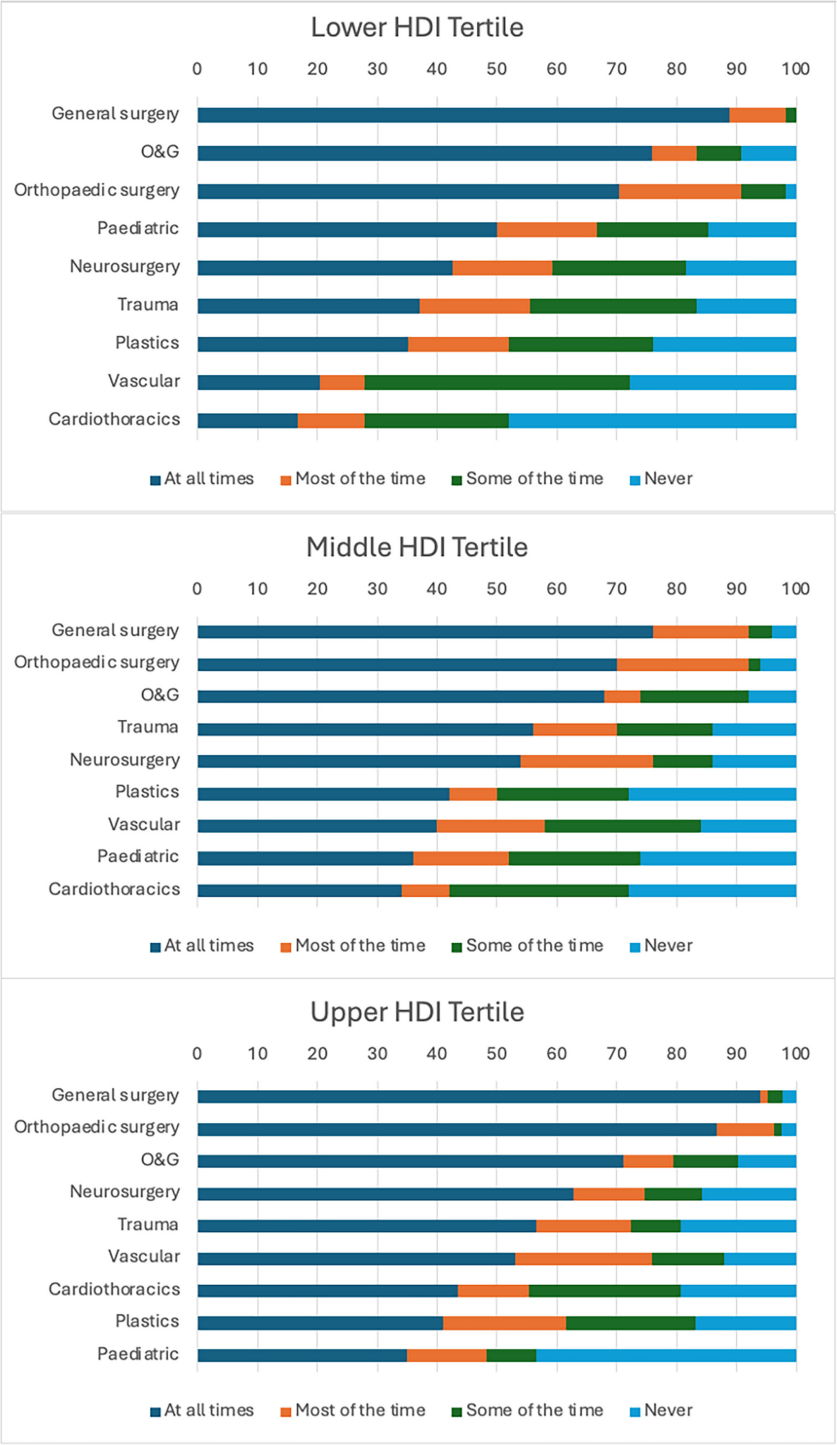
The reported availability of each surgical specialty, stratified by HDI tertile. HDI, Human Development Index; O&G, Obstetrics and Gynaecology

The availability of general surgeons (χ² for trend, p=0.515), orthopaedic surgeons (p=0.171) and obstetricians and gynaecologists (O&G, p=0.676) was equivalent across HDI tertiles (figure 4), when comparing a high availability (present ‘all of the time’ or ‘most of the time’) versus a low availability (present ‘some of the time’ or ‘never’). However, for the surgical subspecialities (paediatric surgery, neurosurgery, trauma surgery, plastic surgery, vascular surgery and cardiothoracic surgery), an increase in high availability was associated with increasing HDI tertile (p<0.001), from 48.2% in the lower HDI tertile to 58.0% in the middle HDI tertile and 64.7% in the upper HDI tertile, respectively (figure 4). Similarly, respondents from upper HDI tertiles reported a high availability of anaesthetists (p=0.003) and intensivists (p<0.001), respectively, compared with the middle HDI tertile and lower HDI tertile.

Respondents were asked to report the number of employed staff and the number of operations performed at their respective hospitals. The ratio of operations performed to general surgeon numbers was the highest in the lower HDI tertile (3.1:1) and middle HDI tertile (3.4:1), compared with the upper HDI tertile (0.9:1, p=0.001). The ratio of operations performed to anaesthetist numbers was also the highest in the lower HDI tertile (7.0:1) and middle HDI tertile (7.6:1), with a 10-fold difference compared with the upper HDI tertile (0.7:1, p=0.006). A higher proportion of patients presenting to lower HDI tertile hospitals (30.0%) were reported to undergo surgery, compared with those presenting to middle (23.0%) and upper (22.3%) HDI tertile hospitals; however, this difference was not statistically significant (p=0.142).

### Post-operative and rehabilitation phase of care

We defined an intensive care unit (ICU) as a unit ‘capable of offering at least mechanical ventilation, or capable of offering both renal replacement therapy and central vasopressor/inotrope administration’ and a high dependency unit (HDU) as a unit ‘capable of offering higher nurse to patient ratios (than the standard hospital inpatient ward) and is capable of offering continuous monitoring of physiology’. There was a high degree of access to an ICU reported by respondents across all strata, with 87.0% in the lower HDI tertile, 98.0% in the middle HDI tertile and 97.6% in the upper HDI tertile. There was more marked variation in the access to an HDU, with significant increase reported from respondents in the lower HDI tertile at 50.0%, through to the middle HDI tertile at 60.0% and the highest in the upper HDI tertile at 72.3% (χ² for trend, p=0.008).

When asked about the main barriers to ICU admission for a patient following a trauma laparotomy, the most common response across all HDI tertiles was limited bed availability. Of note, 16.7% of respondents in the lower HDI tertile described a lack of ICU equipment, such as ventilators, as a barrier; however, this was much less commonly reported by middle HDI tertile and not at all by upper HDI tertile respondents. A proportion of centres reported no barriers to ICU admission, with an increasing proportion from the lower HDI tertile respondents (3 of 54 hospitals, 5.6%) through to upper HDI tertile respondents (25 of 83 hospitals, 22.9%; χ² for trend, p<0.001).

Of the most common causes of postoperative morbidity among patients undergoing a trauma laparotomy, infection was the most commonly cited across all HDI tertiles (online supplemental material). Of note, the lack of resources and nutrition was cited as the second and third most common causes of postoperative morbidity by respondents in the lower HDI tertile.

The availability of physiotherapists (χ² for trend, p<0.001), occupational therapists (p<0.001) and dietitians (p=0.001) all showed increasing availability in the upper HDI tertile, when comparing high availability (present ‘all of the time’ or ‘most of the time’) versus low availability (present ‘some of the time’ or ‘never’). In particular, in the lower HDI tertile, respondents reported a high availability of physiotherapists in 51.9% of cases (28 of 54 respondents), of occupational therapists in 14.8% of cases (8 of 54 respondents) and of dietitians in 35.2% of cases (19 of 54 respondents).

## Discussion

In this global survey of 187 hospitals from 51 countries that provide care for patients receiving trauma care, sizeable discrepancies were clear in the infrastructure, staffing and processes available across the patient pathway. It was evidenced that those in lower resource settings face more financial and procurement-related issues in an attempt to provide optimal trauma care, while those in higher resource settings encounter more system and process-level issues. Hospital-based care also appears to develop the fastest out of the three phases of trauma care, with the challenges of hospital-based care in the middle HDI tertile more analogous to the upper HDI tertile than the lower HDI tertile. However, certain barriers to care appear to be universal across settings, such as delays in prehospital care and the lack of theatre capacity. Obstacles to care highlighted in higher resource settings but not lower resource settings, such as the lack of subspecialist services, should not be seen to be absent in lower resource settings, but rather that presently they are not limiting care to the extent that other factors are. This work highlights clear targets for capacity building in trauma care from across the patient pathway; however, given the breadth of issues highlighted, a systems approach to this problem is essential if the overall standard of global trauma care is to be improved.

Effective prehospital care is essential in delivering good trauma outcomes; trauma is a time-sensitive condition and the timely delivery of definitive management is reliant on efficient prehospital care processes.^28^ Indeed, prehospital care was the phase of care that respondents in the lower HDI tertile reported that needed the most improvement. The lack of formal prehospital care in LMICs has been well described,^9 10 29^ resulting in delays to crucial investigations and interventions. However, such barriers go beyond the actual infrastructure; individuals in many settings will delay seeking initial care due to a variety of societal, cultural or financial factors, instead often seeking alternative routes before engaging with hospital-level care.^30^,^32^ Ultimately, financial barriers remain a great obstacle to accessing trauma care in many low-resource settings, and innovative approaches such as community-based health insurance must be developed to combat this key issue.^33^ Respondents from lower and middle HDI tertiles in our survey highlighted a trio of core concerns in this regard, with a lack of formal emergency medical services, a lack of health education and financial constraints as major barriers to prehospital care, and a recognition of the interplay between these factors is key to designing prehospital care systems in low-resource settings. Moreover, the rural-urban divide in prehospital care must be taken into consideration; rural areas are known to suffer from worse outcomes in trauma across all HDI tertiles^34^,^38^ and long transfer times, as a result of geographical distance or poor road infrastructure, were reported as issues across all tertiles, emphasising the need for equitable resource distribution across regions.

While barriers to effective prehospital care were consistent across lower and middle HDI tertiles, the predominant limitations in intrahospital care among lower HDI tertile hospitals were not seen in either the middle HDI or upper HDI tertile hospitals. Instead, middle HDI tertile centres reported similar issues as upper HDI tertiles, suggesting potentially analogous processes of care and that intrahospital care may have been more consistently invested in, compared to other phases of trauma care. Hospitals are arguably a more attractive target for investment in healthcare than the community, with a hospital being a clearly defined healthcare environment whereby the investment of resources provides a more visible and easily measurable improvement to care, as opposed to any development in the care provided within a community. However, the different phases of care are complementary and investment across all three is needed to maximise the benefit from improvement to any individual phase. Across all HDI tertiles, a lack of operating theatre availability and time to diagnosis were both reported as top causes of delay to laparotomy—the commonality of these factors across settings suggests that they could serve as metrics for benchmarking trauma care in future studies. General surgeons, orthopaedic surgeons and obstetricians play a key role in the management of trauma patients, and encouragingly they were reported to be available to manage trauma patients to a similar degree across tertiles. However, for anaesthetists, subspecialist surgeons and intensivists, this was not the case, with a clear trend in availability with increasing HDI tertile. It should also be noted that task-shifting, of both anaesthesia and surgery, is a key aspect of surgical care in low-resource settings and helps to mitigate the shortage in the healthcare workforce^39^; as an example, a recent study from sub-Saharan Africa found that over half of all operations within a region had been performed by non-specialist physicians.^40^ The provision of subspecialist surgical services should not be equated to the presence of subspecialist surgeons, and the provision of anaesthesia should not be equated to the presence of physician anaesthetists. Also of particular note was the higher number ratios of operations to general surgeons and anaesthetists in the lower and middle HDI tertiles, up to 3-fold and 10-fold respectively, compared with their upper HDI counterparts; this might represent variations in employed doctors per institution, an increased burden of patients, differences in management practice or the availability of diagnostics. Regardless, the severe shortage of surgeons and anaesthetists in the global south is well recognised^5^ and further worsened by migration of physicians from the global south.^41 42^ Therefore, staff retention must be recognised as a key part of trauma system design across all settings.

Infection was the most commonly reported cause of postoperative morbidity across HDI tertiles. If extrapolated to trauma more broadly, this represents a major area of concern across all global populations, especially in the context of rising antimicrobial resistance, with the caveat that cause and treatment of such infections are likely to differ across settings. Moreover, while ICU capabilities were reported to be available across HDI tertiles, a relative lack of HDU availability in the lower and middle HDI tertiles suggests less resilience present in these systems.^43^ The rehabilitation phase of care is an often under-researched aspect of trauma care, yet it was felt to be the phase of care most in need of improvement by respondents in the upper HDI tertile. This was further reinforced by the lack of allied health professionals reported, such as physiotherapists, occupational therapists and dietitians, especially in the lower and middle HDI tertiles.^44^ Many lower resource settings lack appropriate programmes to train these staff,^45^ and improving trauma care standards is an area that requires urgent attention. The economic burden of trauma globally is enormous,^13 14^ and rehabilitation is a key phase of care to ensure that people are able to return to their preinjury functional status and minimise the financial impact of injury at individual, local and national scales.

**Figure 5:**
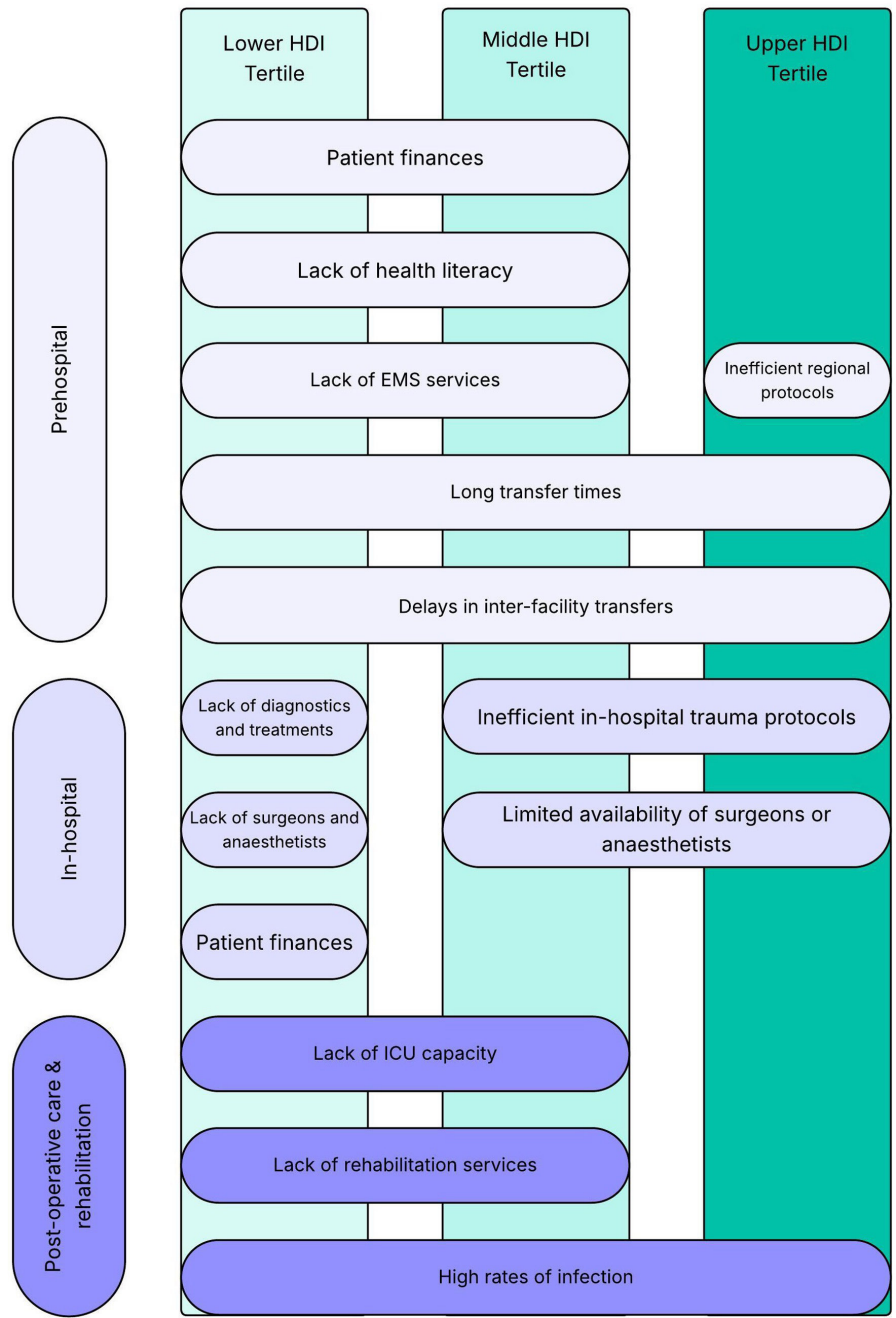
Themes across trauma systems in different HDI tertiles in prehospital, in-hospital and postoperative care and rehabilitation phases of care. EMS, emergency medical serviEmergency Medical Services; HDI, Human Development Index; ICU, intensive care unit.

The opportunity to assess trauma care across a large number of settings with different contexts in terms of resource availability, healthcare staffing and geographical factors provides insight into the development of trauma systems. While previous studies have examined the maturation of trauma systems that have been formally implemented through government policy in high HDI countries,^46 47^ there are few previous literature studies examining the development of heuristic trauma systems in lower resource settings. We have identified a number of common barriers to care between different HDI tertiles across the different phases of trauma care (figure 5). Respondents from lower and middle HDI tertiles reported a number of shared challenges in the delivery of prehospital and rehabilitation care; however, the responses from those in the middle HDI to their intrahospital phase of care suggest their provision here aligns more closely with the upper HDI tertile. Many of the respondents from the middle and upper HDI tertiles cited intrafacility issues and a lack of regional trauma systems as limiting factors for care as they become resource replete, compared with more financial and procurement-related issues raised by the lower HDI tertile respondents. While there is a clear need for investment in healthcare across all phases of care, this cannot be felt to be a panacea for the issues described in this study, as systems-level and process-level issues were clearly evident in more developed regions. Different facets of trauma must be planned and coordinated to ensure that patients feel the maximum benefit of the available healthcare resources.

### Strengths and limitations

This study has surveyed a large number of hospitals that provide trauma care worldwide across a variety of settings, highlighting clear themes and areas for further work. The strengths of this study lie in the diversity of trauma systems its respondents work in, a maximal response rate received and the exploration of the complementary phases of trauma care in these diverse systems. However, the study does come with certain limitations. Many of the participating hospitals were urban and tertiary-level facilities; therefore, caution must be taken in interpreting this work for more rural settings, which are often the most marginalised in healthcare.^5 11 48^ Rural facilities play a key role in the care of injured patients in low-resource settings, and more work is needed to describe how the processes occur in the care of trauma patients in these hospitals. Due to the inclusion criteria of the main study,^18^ certain questions in this survey referred to trauma laparotomy patients specifically, potentially limiting the generalisability of these responses to all-type trauma patients. The care of patients with other types of injuries, such as fractures or traumatic brain injuries, is likely to be influenced by other factors not captured in this survey. Additionally, as the survey was responded to by local clinicians, certain responses may be vulnerable to reporting bias; while this may be suitable for broadly characterising trauma care, it is not a replacement for prospective, granular data that are generated by trauma registries. In order to provide a comparison between resource settings, we have performed a comparison between HDI tertiles; however, each HDI tertile will encompass a vast range of trauma systems that differ significantly in need, access and quality, and such disparities will occur at national, regional and local scales.

## Conclusion

In this study, we have surveyed trauma care across 187 centres in 51 countries and have demonstrated a broad variation in design, implementation and resource availability. Respondents highlighted the challenges in delivering prehospital care and rehabilitation especially in lower and middle HDI tertiles, with intrahospital care appearing to develop preferentially over other phases of care. Certain barriers were ubiquitous across all settings, such as long transfer times, lack of operating theatre availability, delays to diagnosis and postoperative infection, and these common features of trauma care could potentially be used to develop future benchmarks of trauma care. This survey highlights the need for carefully planned investment in trauma care that enables provision of all three phases of care, with coordination of resources to maximise patient benefit.

## Supplementary material

10.1136/bmjgh-2025-021784 online supplemental file 1

## Data Availability

All data relevant to the study are included in the article or uploaded as supplementary information.
