# Supplementary material for: Variation in global trauma care: a survey of 187 hospitals across 51 countries
Source: BMJ Glob Health. 2025 Nov 9;10(11):e021784. doi: 10.1136/bmjgh-2025-021784 (PMC12598983; doi:10.1136/bmjgh-2025-021784)
Supplement: online supplemental file 1 [file bmjgh-10-11-s001.docx]

# Supplementary Material

1 – List of Collaborators

2 – Site Survey Questionnaire

3 – Characteristics of included hospitals

4 – Site Survey Quantitative Data

Figure A1 - Global distribution of recruiting centres, demonstrating number of hospitals enrolled per country

Figure A2 - The most common causes reported by respondents of post-operative morbidity, numbered 1 to 5, stratified by HDI tertile

## Supplementary Material 1 – List of Collaborators

***Writing Group***

T Edmiston, MF Bath, E Cáceres, CM Nuño-Guzmán, DU Baderhabusha, M Khajanchi, J Amoako, K Kohler, AS Hashi, L Carenzo, Z Zhang, M Marsden, R Saleh, C Hammer, L Hobbs, BG Smith, P Hutchinson, TG Weiser, ZB Perkins, TC Hardcastle, T Bashford

***Protocol Development Group***

MF Bath, T Edmiston, BG Smith, D Clarke, A Kwizera, L Hobbs, K Kohler, A Mazzoleni, FFI Fareed, Z Zhang, R Thavayogan, J Erhabor, O Mantle, C Hammer, Z Perkins, M Marsden, R Davenport, RJ Davies, J Amoako, R Moonesinghe, TG Weiser, A Leather, T Hardcastle, R Naidoo, YRA Nordín Servín, A Conway Morris, K Lakhoo, GA Bass, JM Wohlgemut, P Hutchinson, T Bashford

***Hospital Leads***

*Albania*: A Dogjani (University Trauma Hospital; Tirana)

*Algeria*: A Tidjane (EHU-1st November 1954; Oran)

*Argentina*: SE Vélez (Hospital de Urgencias de Córdoba; Córdoba); J Lopez (Hospital de Emergencias Dr Clemente Alvarez; Rosario)

*Australia*: C O’Flynn (The Alfred Hospital; Melbourne)

*Bahrain*: F Haider (Salmaniya Medical Complex Government Hospital; Manama)

*Belarus*: A Litvin (Gomel State Medical University; Gomel)

*Brazil*: R Filho (Hospital Regional Dom Moura; Garanhuns)

*Cameroon*: T Tientcheu (Yaoundé Central Hospital; Yaoundé); S Eya (Centre des Urgence de Yaoundé; Yaoundé)

*Canada*: EG Wong (McGill University Health Centre; Montreal)

*China*: R Wang (Shanghai General Hospital, Shanghai Jiao Tong University School of Medicine, Shanghai); J Wang (Yantai Yuhuangding Hospital, Affiliated with Medical College of Qingdao University; Yantai); Y Ni (The Affiliated Changzhou No.2 People's Hospital of Nanjing Medical University; Changzhou); Z Wang (The People's Hospital of Yuyao City, The Affiliated Yangming Hospital of Ningbo University; Ningbo); Z Tian (Beijing Jishuitan Hospital, Capital Medical University; Beijing); M Fang (Hebei Medical University Third Hospital; Shijiazhuang); M Zhou (The Second Affiliated Hospital of Zhengzhou University; Zhengzhou); S Liu (Zhongda Hospital, Southeast University; Nanjing); K Xie (Tianjin Medical University General Hospital; Tianjin); Z Zhang (Sir Run Run Shaw Hospital, Zhejiang University School of Medicine; Hangzhou); X Guo (Dongguan Eastern Central Hospital, The Sixth Affiliated Hospital of Jinan University; Dongguan); Y Ke (The Second Affiliated Hospital of Kunming Medical University; Kunming); H Ni (Affiliated Jinhua Hospital, Zhejiang University School of Medicine; Jinhua); Q Luo (Shanghai Pudong New Area Gongli Hospital; Shanghai)

*Colombia*: E Caceres (Clínica Universidad de la Sabana; Chía) and LF Reyes (Clínica Universidad de la Sabana; Chía); L Pino (Hospital Universitario del Valle; Cali)

*Democratic Republic of the Congo*: DU Baderhabusha (Hôpital de Kyeshero; Goma); B Cissa Wa Numbe (Hôpital Provincial Général de Référence de Bukavu; Bukavu)

*Egypt*: A Meselhi (Al-Ahrar Zagazig Teaching Hospital; Zagazig); MM Elsayed (Mansoura Specialized Hospital; Mansoura; Delta Hospital; Mansoura); S Abdelmohsen (Aswan University Hospital; Aswan); A Elkhouly (Tanta University Hospital; Tanta); A Elmorshdy (Mataria Teaching Hospital; Cairo); G Abouelnagah (Alexandria Main University Hospital; Alexandria); A Osman (Beni Suef Health Insurance Hospital; Beni Suef); H Taher (Kasr Al Ainy Hospital; Cairo)

*Ethiopia*: W Shenkutie (Arsi University Asella Referral and Teaching Hospital; Asella); M Senbu (Adama Hospital Medical college; Adama City); N Bayleyegn (Jimma University Medical Centre; Jimma); M Merene (ALERT Comprehensive Specialized Hospital; Addis Ababa); M Ahmed (St. Paul’s Hospital Millenium Medical College; Addis Ababa)

*Ghana*: S Gudugbe (Holy Family Catholic Hospital; Techiman); J Amoako (Korle Bu Teaching Hospital; Accra); M Morna (Cape Coast Teaching Hospital; Cape Coast); E Gyabaah (Sunyani Teaching Hospital; Sunyani); H Seidu-Aroza (Ho Teaching Hospital; Ho)

*Greece*: I Baloyiannis (General University Hospital of Larissa; Larissa); K Bouchagier (University Hospital of Patras; Patras); F Mulita (General Hospital of Aigio; Aigio); A Ioannidis (AHEPA University Hospital of Thessaloniki; Thessaloniki)

*Guatemala*: M Rivera (Hospital General San Juan de Dios; Guatemala City)

*Haiti*: F Régis (Hôpital Universitaire La Paix; Port-au-Prince)

*India*: L Bains (Maulana Azad Medical College; New Delhi); M Khajanchi (Seth GS Medical College and KEM Hospital; Mumbai)

*Italy*: L Sartarelli (Presidio Ospedaliero Centrale - SS. Annunziata; Taranto); R Bollino (Azienda USL - IRCCS di Reggio Emilia; Reggio Emilia); M Fedi (San Jacopo Hospital, Pistoia); A Bottari (Ospedale S. Maria alla Gruccia; Montevarchi); F Cammelli (Azienda Ospedaliero Universitaria Careggi; Firenze); G Calini (IRCCS Azienda Ospedaliero - Universitaria di Bologna; Bologna); A Piccolo (Grande Ospedale Metropolitano; Reggio Calabria); D Visconti (AOU Cittá della Salute e della Scienza; Torino); M Altomare (ASST Grande Ospedale Metropolitano Niguarda; Milan); L Carenzo (IRCCS Istituto Clinico Humanitas; Milan); F Fleres (AOU Policlinico G Martino; Messina)

*Japan*: Y Iwao (Ohta Nishinouchi Hospital; Fukushima)

*Kenya*: R Parker (Tenwek Hospital; Tenwek)

*Malaysia*: CK Tiong (Universiti Malaya Medical Centre; Kuala Lumpur); CX Teoh (Hospital Canselor Tuanku Muhriz UKM; Kuala Lumpur); AD Zakaria (USM Specialist Hospital, Universiti Sains Malaysia; Kubang Kerian)

*Mexico*: CM Nuño-Guzmán (Hospital Civil de Guadalajara Fray Antonio Alcalde; Guadalajara); A González-Ojeda (Centro Médico Nacional de Occidente IMSS; Guadalajara)

*New Zealand*: C Wakeman (Christchurch Hospital; Christchurch)

*Niger*: E Ikwutah (SIM Galmi Hospital; Galmi)

*Nigeria*: M Daniyan (Ahmadu Bello University Teaching Hospital; Zaria); A Adamu (Abubakar Tafawa Balewa University Teaching Hospital; Bauchi); E Akpo (Delta State University Teaching Hospital; Oghara); I Chukwu (Federal Medical Centre; Umuahia); M Bashiru (Federal Medical Center Nguru; Nguru); B Akanni (Alex Ekwueme Federal University Teaching Hospital; Abakaliki); J Olaogun (Ekiti State University Teaching Hospital; Ado Ekiti); B Nomayo-Oriabure (The Hills Medical Center; Benin City); H Abiyere (Federal Teaching Hospital Ido-Ekiti; Ido Ekiti); E Oriabure (University of Benin Teaching Hospital; Benin City)

*Pakistan*: FF Khidri (Liaquat University of Medical and Health Sciences; Jamshoro); SA Naqi (Indus Hospital and Health Network; Karachi); T Khan (Lady Reading Hospital; Peshawar); K Faheem (PAF Hospital Base Faisal; Karachi)

*Paraguay*: RS Pederzoli (Hospital de Trauma Prof. Dr. Manuel Giagni; Asunción)

*Occupied Palestinian Territories*: H Abu-Arish (Al-Ahli Hospital and Hebron Governmental Hospital; West Bank); M Youssef (Nasser Hospital; Gaza)

*Peru*: C Huaroto-Landeo (Clinica Internacional, Lima)

*Portugal*: I Carolino Gomes (Unidade Local de Saúde de Lisboa Ocidental; Lisboa); N Gatta (Unidade Local de Saúde São João; Porto)

*Romania*: I Negoi (Clinical Emergency Hospital of Bucharest; Bucharest)

*Russia*: S Katorkin (Clinics of Samar Medical University; Samara)

*Saudi Arabia*: N Alsubaie (King Saud University Medical City; Riyadh)

*Singapore*: JTT Goo (Khoo Teck Puat Hospital; Singapore); S Balasubramaniam (Tan Tock Seng Hospital; Singapore)

*Somalia*: MS Hassan (Mogadişu Somali-Türkiye Recep Tayyip Erdoğan Training and Research Hospital; Mogadishu); SA Mohamed (Kakaal Hospital; Mogadishu); AE Abdishakur (Somali-Sudanese Specialized Hospital; Mogadishu)

*South Africa*: TC Hardcastle (Inkosi Albert Luthuli Central Hospital; Durban); R Naidoo (Ngwelezana Hospital; Empangeni); R Crawford (Chris Hani Baragwanath Academic Hospital; Johannesburg); M Moeng (Charlotte Maxeke Johannesburg Academic Hospital; Johannesburg); HJ Kruger (Tygerberg Hospital; Cape Town)

*Spain*: M Serrano-Navidad (Hospital General Universitario de Elche; Alicante); A Landaluce-Olavarria (Urduliz Hospital; Bizkaia); CC Lopes Moreira (Hospital Universitario Donostia; San Sebastián); H Llaquet-Bayo (Hospital Universitari Parc Taulí; Sabadell)

*Sri Lanka*: K Jayasuriya (District General Hospital Kegalle; Kegalle); JASB Jayasundara (District General Hospital; Dambulla); D Subasinghe (National Hospital of Sri Lanka; Colombo); J Mithushan (Teaching Hospital Batticaloa; Batticaloa)

*Sudan*: A Ibrahim (Atbara Teaching Hospital; Atbara); M Elnour (Prince Digna Referral Hospital; Port Sudan); I Ahmed (Al Hasahissa Teaching Hospital; Al-Hasahisa); I Adel (Bashair Teaching Hospital; Khartoum); L Mohammed (Port Sudan Teaching Hospital; Port Sudan); S Bakhit (Dongola Specialized Hospital; Dongola); M Elbashier (Kassala Teaching Hospital; Kassala City); R Musa (Alnao Teaching Hospital; Omdurman City); J Amin (Sinnar Teaching Hospital; Sinnar City); M Yassin (Gadarif Teaching Hospital; Gadarif); A Babiker (Ad-Damazin Teaching Hospital; Ad-Damazin); A Noureldin (New Halfa Teaching Hospital; New Halfa); A Abdalazeez (Zalingei Teaching Hospital; Zalingei City)

*Sweden*: S Benediktsdottir (Skåne Universitetssjukhus Malmö; Malmö)

*Syria*: L Hasan (Damascus Hospital; Damascus); S Hamad (Al Mouwasat Hospital; Damascus); N Mansour (Homs University Hospital; Homs)

*Thailand*: O Homchan (Maharaj Nakorn Chiang Mai Hospital; Chiang Mai)

*Tunisia*: A Hasnaoui (Menzel Bourguiba Hospital; Bizerte); A Bouzid (Mahmoud Matri Hospital; Ariana); W Riahi (Beja Hospital; Beja)

*Türkiye*: M Ergenç (Marmara University School of Medicine; Istanbul); B Yigit (Bagcilar Training and Research Hospital; Istanbul); B Citgez (Uskudar University Faculty of Medicine, Memorial Hospital; Istanbul); M Yilmaz (Kocaeli City Hospital; Izmit); YF Aydoğdu (Bandirma Training and Research Hospital; Balıkesir); A Guner (Karadeniz Technical University Faculty of Medicine, Farabi Hospital; Trabzon); H Karakullukcu (Sultan Abdülhamid II Han Training and Research Hospital; Istanbul); K Tuncer (Bakircay University Cigli Education and Research Hospital; Izmir); AN Sanli (Private ADN International Hospital; Gaziantep); MT Demirpolat (University of Health Sciences Umraniye Training and Research Hospital; Istanbul); Ç Büyükkasap (Gazi University Hospital; Ankara); AC Yildirim (Kütahya City Hospital; Kütahya); F Feratoglu (Sultanbeyli State Hospital; Istanbul)

*Ukraine*: S Smoliar (Kharkiv Regional Clinical Hospital; Kharkiv)

*United Kingdom*: H Roocroft (Southmead Hospital; Bristol); G McKnight (University Hospital of Wales, Cardiff); M Hughes (Royal London Hospital; London); JV Taylor (University Hospital Aintree; Liverpool); E Yung (Aberdeen Royal Infirmary; Aberdeen); EJ Nevins (Sunderland Royal Hospital; Sunderland); S Owen-Smith (University Hospital Plymouth; Plymouth); A Mian (John Radcliffe Hospital; Oxford); M Alfa-Wali (St Mary’s Hospital; London); C Menichetti (Queen Elizabeth Hospital; Birmingham); T Jodlowski (Salford Royal Hospital; Salford); S Mundell (Morriston Hospital; Swansea); O Khalil (Norfolk and Norwich University Hospital; Norwich); S Jay (Addenbrooke’s Hospital; Cambridge); M El-Boghdady (St George’s University Hospital; London); P Pratheepan (North Middlesex University Hospital; London); A Abouelnaga (Manchester Royal Infirmary; Manchester); A Brooks (Nottingham University Hospitals; Nottingham); S Yoong (Royal Victoria Hospital; Belfast); Y Al Azzawi (Royal Infirmary of Edinburgh; Edinburgh); YS Lim (Dr Gray’s Hospital; Elgin)

*United States*: S Agarwal (Duke University Hospital; Durham); P Petrone (NYU Langone Hospital–Long Island; New York); D Stephens (Mayo Clinic; Rochester); N Starr (Zuckerberg San Francisco General Hospital; San Francisco); A Teichman (Rutgers RWJ Hospital; New Brunswick); C Dodgion (Froedtert & the Medical College of Wisconsin; Milwaukee); C Wolff (Cleveland Clinic Akron General; Akron); T Egodage (Cooper University Hospital; Camden); J Brady (Chippenham Hospital; Richmond); J Brown (University of Pittsburgh Medical Center; Pittsburgh); D Leon (University of California Davis Medical Center; Sacramento)

*Uruguay*: A Pienovi (Hospital de Clínicas Dr. Manuel Quintela; Montevideo)

*Yemen*: R Saleh (Al Thawrah Hospital Ibb; Ibb)

***Collaborators***

*Albania*: K Doçi, E Bregaj (University Trauma Hospital; Tirana)

*Algeria*: J Mansouri, B Tabeti (EHU-1st November 1954; Oran)

*Argentina*: M Titarelli, MM Avalos Barraza , M Sánchez (Hospital de Urgencias de Córdoba; Córdoba); E Caldani, A Giavarini (Hospital de Emergencias Dr Clemente Alvarez; Rosario)

*Australia*: C Groombridge, E Ban (The Alfred Hospital; Melbourne)

*Bahrain*: A Abdulla (Salmaniya Medical Complex Government Hospital; Manama)

*Belarus*: V Bereshchenko, P Tereshchenko (Gomel State Medical University; Gomel)

*Brazil*: I Marcos, R Lima (Hospital Regional Dom Moura; Garanhuns)

*Cameroon*: N Nwenasi (Yaoundé Central Hospital; Yaoundé); E Aloys (Centre des Urgence de Yaoundé; Yaoundé)

*Canada*: H Uchino, JR Grushka, W Davalan (McGill University Health Centre; Montreal)

*China*: C Chen, F Ge (Shanghai General Hospital, Shanghai Jiao Tong University School of Medicine, Shanghai); K Lu (The Affiliated Changzhou No.2 People's Hospital of Nanjing Medical University; Changzhou); J Zhang (The People's Hospital of Yuyao City, The Affiliated Yangming Hospital of Ningbo University; Ningbo); X Liu, X Li (Beijing Jishuitan Hospital, Capital Medical University; Beijing); Z Qi (Hebei Medical University Third Hospital; Shijiazhuang); N Wang, J Tang, S Wang, F Gao, Y Lu, H Du (The Second Affiliated Hospital of Zhengzhou University; Zhengzhou); C Wu, H Fu (Zhongda Hospital, Southeast University; Nanjing); J Liu (Tianjin Medical University General Hospital; Tianjin); T Chen (Sir Run Run Shaw Hospital, Zhejiang University School of Medicine; Hangzhou); M Du, J Guan (Dongguan Eastern Central Hospital, The Sixth Affiliated Hospital of Jinan University; Dongguan); Q Lu, Y Li, J Li, Q Wang, W Luo (The Second Affiliated Hospital of Kunming Medical University; Kunming); K Wang (Affiliated Jinhua Hospital, Zhejiang University School of Medicine; Jinhua); H Zhang, J Dong (Shanghai Pudong New Area Gongli Hospital; Shanghai)

*Colombia*: S Gelvez, K Reyes (Hospital Universitario del Valle; Cali)

*Democratic Republic of the Congo*: D Tsongo, J Muhoza (Hôpital de Kyeshero; Goma); A Mirindi, JR Birindwa (Hôpital Provincial Général de Référence de Bukavu; Bukavu)

*Egypt*: H Aboelfadl, A Elasad, S Elsheikh, F Elsaied, M Atef, R Elnour, N Elbaloula, EA Shanab, M Adres, O Mohamed, S Adam (Al-Ahrar Zagazig Teaching Hospital; Zagazig); O Younes, T Elboraay, O Abdelfattah, MA Elfadali, A Jader, A Ziada, K Sarhan, M Sherif , R Gomaa, R Mohamed, E Fouda, A Elshaboury, M Alshraiedeh, H Elhadidi, A Eldiasti, M Ahmed, E Elkoury, H Abdelhady, M Amasha, A Elbadrawy, M Nassif, M Hamed (Mansoura Specialized Hospital; Mansoura); D Zahran, S Abdelaal, OS Abdelfattah, W Shehada, M Alsharif, M Elnadi, O Sharaf, M Elgliand, M Badr, A Hegazi, A Gohar, A Elshal, M Abdelhady, M Saadawi, M Mohammed, EY Salem (Delta Hospital; Mansoura); M Madany (Aswan University Hospital; Aswan); H Mansour, A Ashour, A AbuSuliman, K Tolba, MS Elgendy, M Ezz, M Marei, HI Taha, I Younes, A Abouammar (Tanta University Hospital; Tanta); R Wael, Asmaa Elmorshdy, Aesha Elmorshdy, AM Ibrahim, A El-Borollosy, S Elmorshdy, A Mohamed, A Adel, NH El-Saeed, M Essam, EAK Abdelraheem, M Shaapan, M Salah, E Saber, MA Ibrahim, R Mamdouh, AMM Mohamadin, F Moharb (Mataria Teaching Hospital; Cairo); O Shaqran, Z Selim, Y Tanas, M Khalil, B Eldin, Y Gaber, A Ibrahim, D Bekhit, B Mohamed, A Farrag (Alexandria Main University Hospital; Alexandria); M Saadawi, H Mahfouz (Beni Suef Health Insurance Hospital; Beni Suef); N Sayed, M El Mahrouki, A Amgad, D Elmagdoub, S Paulo (Kasr Al Ainy Hospital; Cairo)

*Ethiopia*: B Gebremedhin, A Eticha (Arsi University Asella Referral and Teaching Hospital; Asella); B Bayissa, K Urgessa, B Tasew (Adama Hospital Medical college; Adama City); Y Yilma, LG Mude, O Tilahun (Jimma University Medical Centre; Jimma); L Buta, AG Mideksa, T Gemechu (ALERT Comprehensive Specialized Hospital; Addis Ababa); A Tilahun, Y Degefu (St. Paul’s Hospital Millenium Medical College; Addis Ababa)

*Ghana*: F Quenin, E Acquah, I Abdull-Karim, C Takyi (Holy Family Catholic Hospital; Techiman); G Aryee, T Wordui, A Bowan, P Kumassah, N Adu-Aryee, N Naalane, F Dedey, J Nsaful (Korle Bu Teaching Hospital; Accra); O Ekor, G Rahman, M Nortey, R Baidoo, M Amoako-Boateng, D Enti, K Agyen-Mensah, E Quartson, T Agyen, E Ofori, P Mensah, V Kudoh, D Arthur, P Maison (Cape Coast Teaching Hospital; Cape Coast); F Akum, F Owusu (Sunyani Teaching Hospital; Sunyani); N Affram, D Tamatey (Ho Teaching Hospital; Ho)

*Greece*: C Sarakatsianou, D Papaspyrou (General University Hospital of Larissa; Larissa); A Antzoulas, K Kitsou, V Garantzioti, (University Hospital of Patras; Patras); V Leivaditis (General Hospital of Aigio; Aigio); A Vouchara, K Katsiafliaka (AHEPA University Hospital of Thessaloniki; Thessaloniki)

*Guatemala*: S Morales, E Galindo, A Meza, M Colón, E Cardona (Hospital General San Juan de Dios; Guatemala City)

*Haiti*: K Louis, R Osias, C Lominy, A Capois (Hôpital Universitaire La Paix; Port-au-Prince)

*India*: SA Khan, V Verma (Maulana Azad Medical College; New Delhi); S Amin, A Gaikwad (Seth GS Medical College and KEM Hospital; Mumbai)

*Italy*: V Tonini, M Cervellera (Presidio Ospedaliero Centrale - SS. Annunziata; Taranto); M Fumagalli, M Zizzo, D Luppi, H Yu, L Di Donato (Azienda USL - IRCCS di Reggio Emilia; Reggio Emilia); F Leo, C Cecchi, G Ripamonti, B Pesi, L Piombetti, M Pagani, G Pascale, S Di Salvatore, C Tasca, S Giannessi, R De Vincenti, E Monati (San Jacopo Hospital, Pistoia); F Renzi (Ospedale S. Maria alla Gruccia; Montevarchi); L Vacca, F Matarazzo, D Perini, A Di Bella, L Fortuna (Azienda Ospedaliero Universitaria Careggi; Firenze); M Rottoli, M Binetti (IRCCS Azienda Ospedaliero - Universitaria di Bologna; Bologna); M Tescione, G Sera, N Pellicano, S Pangallo (Grande Ospedale Metropolitano; Reggio Calabria); E Ballauri, M Santarelli (AOU Cittá della Salute e della Scienza; Torino); S Cimbanassi, S Cioffi, G Curreri (ASST Grande Ospedale Metropolitano Niguarda; Milan); M Ceolin, D Del Fabbrio, S Giudici, M Cecconi (IRCCS Istituto Clinico Humanitas; Milan); T Sinicropi, C Mazzeo (AOU Policlinico G Martino; Messina)

*Japan*: K Sato (Ohta Nishinouchi Hospital; Fukushima)

*Kenya*: K Otoki, D Baraka (Tenwek Hospital; Tenwek)

*Malaysia*: RMZ Ang, MNF Zulkifli, KC Sheng, MTY Wong, N Aziz, PJH Lim, CCE Koay (Universiti Malaya Medical Centre; Kuala Lumpur); YX Teoh, I Chik (Hospital Canselor Tuanku Muhriz UKM; Kuala Lumpur); Z Zakaria, MHS Satar, MR Mazlan (USM Specialist Hospital, Universiti Sains Malaysia; Kubang Kerian)

*Mexico*: L Bravo-Cuéllar, J Orozco-Camacho, A Nava-Franco, M Ibarra-Tapia, F López-Ortega, F Romo-Pérez, R Contreras-Arias, M Alejo-Rivera (Hospital Civil de Guadalajara Fray Antonio Alcalde; Guadalajara); SJ Vázquez-Sánchez, C Fuentes-Orozco (Centro Médico Nacional de Occidente IMSS; Guadalajara)

*New Zealand*: A McCombie, J Dasril, Y Teo (Christchurch Hospital; Christchurch)

*Niger*: AA Fagbenro, K Shafer (SIM Galmi Hospital; Galmi)

*Nigeria*: L Iji, S Gana, M Bashir, A Ajayi, I Gundu, GD Mukoro, L Ukwubile, C Okeke, A Jimoh, V Nduka (Ahmadu Bello University Teaching Hospital; Zaria); K Bwala, A Ningi (Abubakar Tafawa Balewa University Teaching Hospital; Bauchi); S Oriakhi, H Odion-Obomhense (Delta State University Teaching Hospital; Oghara); S Ekpemo, K Okpokiri (Federal Medical Centre; Umuahia); AA Makama, H Aminu, Z Ahmad, M Mustapha, O Segunfunmi (Federal Medical Center Nguru; Nguru); E Boladuro, U Eni, C Obi, NL Kwentoh (Alex Ekwueme Federal University Teaching Hospital; Abakaliki); D Idowu, M Magbagbeola (Ekiti State University Teaching Hospital; Ado Ekiti); O Adolphus, E Oriabure (The Hills Medical Center; Benin City); S O Fatudimu; O Oloruntoba (Federal Teaching Hospital Ido-Ekiti; Ido Ekiti); R Eghonghon, S Omorogbe (University of Benin Teaching Hospital; Benin City)

*Pakistan*: S Shaikh, AK Narsani, I Ujjan, A Munir, AI Memon, F Hameed, S Khatoon, A Talpur, S Kumar, ZA Yousfani, N Dal, S Naz, M Akbar, AM Bhatti, N Amir, SA Khaskheli (Liaquat University of Medical and Health Sciences; Jamshoro); N Iqbal, A Aamir, G Shamsi, G Awais, I Tasleem, N Iodhi, I Ahmed, R Fatima, S Asif, H Haroon, A Jawaid, J Muneer, H Ahmed, M Washdil, A Hilal, M Ishaq, S Ialani, Y Kumar, MN Shehzad, S Nadeem, N Ahmed, S Ahmed, S Gulzar (Indus Hospital and Health Network; Karachi); G Khan, Z Jaffer, A Gul, M Khan, A Faraz, M Obaid (Lady Reading Hospital; Peshawar); H Pirhay, K Faheem, M Shafique, R Nafees (PAF Hospital Base Faisal; Karachi)

*Paraguay*: LC Soares Barboza de Toledo, AL Silva de Sousa (Hospital de Trauma Prof. Dr. Manuel Giagni; Asunción)

*Occupied Palestinian Territories*: B Qneiby, R Jabari, R Farash (Al-Ahli Hospital; West Bank); M Oweidat (Hebron Governmental Hospital; West Bank); R Matar, M Shaldan (Nasser Hospital; Gaza)

*Peru*: C Picasso-Arias (Clinica Internacional, Lima)

*Portugal*: C Strong, F Feliciano, L dos Santos, D Monteiro (Unidade Local de Saúde de Lisboa Ocidental; Lisboa); S Alves, D da Cruz (Unidade Local de Saúde São João; Porto)

*Romania*: B Oprita, E Dumitru (Clinical Emergency Hospital of Bucharest; Bucharest)

*Russia*: L Lichman, O Davydova, P. Andreev (Clinics of Samar Medical University; Samara)

*Saudi Arabia*: R Alyahya, AM Alrwais, NH Almadi, N AlShahwan, M Aladawi, SH Aldeligan, A Alotaibi (King Saud University Medical City; Riyadh)

*Singapore*: J Lee, S Gunasekaran, MW Ong, DJK Lee, WW Lim (Khoo Teck Puat Hospital; Singapore); LT Teo, RL Tan (Tan Tock Seng Hospital; Singapore)

*Somalia*: AS Hashi, AA Omar, AN Mohamed, AM Abdi (Mogadishu Somali-Türkiye Recep Tayyip Erdoğan Training and Research Hospital, Mogadishu); AH Salad (Kakaal Hospital; Mogadishu); AE Abdishakur (Somali-Sudanese Specialized Hospital; Mogadishu)

*South Africa*: F Ganchi, S Naidoo, K Moodley, H Wain (Inkosi Albert Luthuli Central Hospital; Durban); N Reddy (Ngwelezana Hospital; Empangeni); N Laher, D Wineberg, R Pretorius, R Pswarayi, E Laney, O Lusawana, A Mushtaq, I Bogiages, F Viljoen (Chris Hani Baragwanath Academic Hospital; Johannesburg); F Mohammed, G Jacks, L Mohlala, C Nyatsambo, S Mathibela, A Nortje, S Makhadi, T Pratt, K de Kock (Charlotte Maxeke Johannesburg Academic Hospital; Johannesburg); MQ Patel, M Parker, JJP Buitendag, GV Oosthuizen (Tygerberg Hospital; Cape Town)

*Spain*: C Martínez de Carneros, S Quinto Llopis (Hospital General Universitario de Elche; Alicante); L Cruzado, A Sainz-Lete, B Estraviz-Mateos, JC Zevallos-Quiroz (Urduliz Hospital; Bizkaia); I Augusto Ponce, A Garcia Domínguez, A Lizarazu Perez, A Rodriguez Gonzalez (Hospital Universitario Donostia; San Sebastián); A Muñoz-Campaña, A Campos-Serra (Parc Taulí Hospital Universitari; Sabadell)

*Sri Lanka*: L Bandara, K Gunasekara, G Jayarathne, Y Arachchi, M Priyangani, S Wimalge (District General Hospital Kegalle; Kegalle); RSC Desman, K Gunarathne (District General Hospital; Dambulla); G Wimalasena, V Rohana, S Ranathunga (National Hospital of Sri Lanka; Colombo); S Harikrishanth, J Jeyaruban (Teaching Hospital Batticaloa; Batticaloa)

*Sudan*: M Mohammed, A Mohamed, L Zeinalabedeen, A Mahmoud (Atbara Teaching Hospital; Atbara); M Mohamed (Prince Digna Referral Hospital; Port Sudan); M Eltahir, G Ahmed, M Ahmed (Al Hasahissa Teaching Hospital; Al-Hasahisa); IAO Mohammed, SAA Ibrahim, EAH Aziz (Bashair Teaching Hospital; Khartoum); M Homida, FS Mahdi (Port Sudan Teaching Hospital; Port Sudan); M Issak, A Mohammed (Dongola Specialized Hospital; Dongola); M Hafiz, H Makki (Kassala Teaching Hospital; Kassala City); N Awad, A Elhassan (Alnao Teaching Hospital; Omdurman City); M Amin, A Daffalla, A Omer (Sinnar Teaching Hospital; Sinnar City); M Alhadi, M Mostafa, O Eljizoly (Gadarif Teaching Hospital; Gadarif); A Musa, M Abdallah (Ad-Damazin Teaching Hospital; Ad-Damazin); G Fakhri, A Ahmed, A Mohammed (New Halfa Teaching Hospital; New Halfa)

*Sweden*: M Kollind, S Marchesi (Skåne Universitetssjukhus Malmö; Malmö)

*Syria*: A Aldirani, A Almahjaa, M Abdulkareem, E Kallas, A Alfandi, A Hejazi, B Alnaser, A Alnaser, J Sandouk (Damascus Hospital; Damascus); S Sara, K Ballan, H Joha (Al Mouwasat Hospital; Damascus); S Kassis, W Abboud, M Ahmad, A Hamdan (Homs University Hospital; Homs)

*Thailand*: K Chandacham, T Jirapongcharoenlap, N Chotirosniramit (Maharaj Nakorn Chiang Mai Hospital; Chiang Mai)

*Tunisia*: R Trigui, O Gaidi (Menzel Bourguiba Hospital; Bizerte); A Saidani, A Belhaj (Mahmoud Matri Hospital; Ariana); H Zebda, A Menif, R Khelili (Beja Hospital; Beja)

*Türkiye*: Ç Bayır, Ö Acar, E Bozlakoğlu (Marmara University School of Medicine; Istanbul); E Yavuz, G Alici, S Meric, N Bugdayci, A Sayar, A Ergin, A Saylar, A Barcin, Y Altinel, O Gulcicek, O Cakir (Bagcilar Training and Research Hospital; Istanbul); H Ozsahin, C Ersavas (Uskudar University Faculty of Medicine, Memorial Hospital; Istanbul); GK Aydoğdu (Bandirma Training and Research Hospital; Balıkesir); K Saraçoğlu, N Dundar (Kocaeli City Hospital; Izmit); K Eyuboglu, R Tekcan, M Bodur, M Aktas, B Erdem, A Calik, A Kodalak, A Oruc, M Usta, B Alkas, M Rahimi, A Cekic, D Pehlivan, I Rizaoglu, A Mwinyi, B Canakci, M Shehada, S Topaloglu (Karadeniz Technical University Farabi Hospital; Ortahisar); A Karaaslan, G Ercan, Y Poyrazoğlu, M Çuhadar, Ö Özkan (Sultan Abdülhamid II Han Training and Research Hospital; Istanbul); R Ağcabay, G Tuncer, S Farsak, C Tuğmen, N Polat, E Kebapçı, M Yıldırım, N Göret, M Gündal, S Ünlü, E Tekel (Bakircay University Cigli Education and Research Hospital; Izmir); A Özpek, H Tosun (University of Health Sciences Umraniye Training and Research Hospital; Istanbul); B Yeşilova, K Dikmen, H Göbüt, A Yavuz (Gazi University Hospital; Ankara); S Zeren, Y Sönmez (Kütahya City Hospital; Kütahya); T Gulsen, M Zenciroglu (Sultanbeyli State Hospital; Istanbul)

*Ukraine*: P Kyrylo, P Kostiantyn, P Ivan (Kharkiv Regional Clinical Hospital; Kharkiv)

*United Kingdom*: P Orchard, J Fyfe (Southmead Hospital; Bristol); H Dowell, O Braun, M Creed, P Strong, F Sweeney, N Mitchell, I McClure, D Parry, O Gbadegesin, E Carrington-Windo, M McKenna, S Mundell, L Hall, S Gasson, E Crudge, A Eglinton (University Hospital of Wales, Cardiff); R Davenport, P Vulliamy, ZB Perkins, O Ugas (Royal London Hospital; London); L Holt, H Jenkinson, J Tan (University Hospital Aintree ; Liverpool); G Ramsay, O Adepoju, R Cummine, S Tariq, A Mohammad, L Wilson (Aberdeen Royal Infirmary; Aberdeen); A Musbahi, R Coates (Sunderland Royal Hospital; Sunderland); SJ Horne, N Preda, F Luvisetto (University Hospital Plymouth; Plymouth); Z Zhang, I Saqib, G Matzakanis, P Pearce (John Radcliffe Hospital; Oxford); N Sánchez-Thompson, C Scurr, A Bernstein (St Mary’s Hospital; London); MS Gonsalves, MS Hoque-Uddin, I Abbott, O Dada (Queen Elizabeth Hospital; Birmingham); S Jamil, H Read, D Horner, R Doonan, A Stafford (Salford Royal Hospital; Salford); C Battle (Morriston Hospital; Swansea); R Thavayogan, O Quinn (Norfolk and Norwich University Hospital; Norwich); M Powar, S Ling, S Gourgiotis (Addenbrooke’s Hospital; Cambridge); H Shinwari (St George’s University Hospital; London); S Mohandas (North Middlesex University Hospital; London); M Eldoadoa, R Ismail (Manchester Royal Infirmary; Manchester); G Melia, N Gandhi, L Blackburn (Nottingham University Hospitals; Nottingham); T Merchant (Royal Victoria Hospital; Belfast); J Robinson, S Mackie (Royal Infirmary of Edinburgh; Edinburgh); YS Wong, Q Lee (Dr Gray’s Hospital; Elgin)

*United States*: D Moris, CP Nicholson Jr., S Provencher, J Cook (Duke University Hospital; Durham); G Baltazar, K Cordero-Bermudez (NYU Langone Hospital - Long Island; New York); LE Walker, MK Abou Chaar (Mayo Clinic; Rochester); R Koch, K Faktor, A Chang (Zuckerberg San Francisco General Hospital; San Francisco); Z Englert, C Kyaw, N Pirozzi, L Moko, B Chernock, E Marshall (Rutgers RWJ Hospital; New Brunswick); JA Gellings (Froedtert & the Medical College of Wisconsin; Milwaukee); J Krizo, J Molinari (Cleveland Clinic Akron General; Akron); E Hancin, I Armento (Cooper University Hospital; Camden); P Hu, R Uhlich, E Barnes (Chippenham Hospital; Richmond); A Rawal, O Falade (University of Pittsburgh Medical Center; Pittsburgh); D Nishijima, D Leshikar (University of California Davis Medical Center; Sacramento)

*Uruguay*: E Delgado (Hospital de Clínicas Dr. Manuel Quintela; Montevideo)

*Yemen*: S Al Wageeh, A Al Yafrosi (Al Thawrah Hospital Ibb; Ibb)

***Data Validators***

*Haiti*: L Minthor (Hopital Universitaire La Paix, Port-au-Prince)

*Italy*: S Cardelli (IRCCS Azienda Ospedaliero - Universitaria di Bologna, Bologna)

*Malaysia*: W Chiew Meng (Universiti Malaya Medical Centre, Kuala Lumpur); S Johan (USM Specialist Hospital, Universiti Sains Malaysia; Kubang Kerian)

*Nigeria*: IE Ihedoro (Federal Medical Centre, Abia)

*Pakistan*: A M Waryah (Liaquat University of Medical and Health Sciences, Jamshoro)

*Peru*: D Chavez (Clinica Internacional, Lima)

*Sri Lanka*: T Manivannan (Teaching Hospital Batticaloa, Batticaloa)

*Sudan*: M A M Abdalla (Dongola Specialized Hospital, Dongola)

*Tunisia*: A Itaimi (Menzel Bourguiba Hospital, Bizerta)

*Türkiye*: TK Uprak (Marmara University School of Medicine, Istanbul); M Ulusahin (Karadeniz Technical University Farabi Hospital, Trabzon)

*United Kingdom*: CV Riley (Salford Royal Hospital, Salford); H Hussein (North Middlesex University Hospital, London); T Edmiston (Addenbrookes Hospital, Cambridge); L Nicol (Dr Gray's Hospital, Elgin)

## Supplementary Material 2 – Site Survey Questionnaire

Hospital Characteristics and Provision

Would you define the current healthcare facility you work in as urban or rural?

- Urban

- Rural

Please specify the type of healthcare facility you currently work in

- Public / Government led

- Private (for profit)

- Private (not for profit, e.g. mission hospital)

How is patients' trauma care funded for at your healthcare facility? Please select all that apply

- Direct government funded (full cover)

- Direct government funded (subsidised / partial cover)

- Patient-specific health insurance

- Patient out-of-pocket expenditure

- Charity-funding or non-governmental organisation (NGO)

- Other (please specify)

How would you define the type of healthcare facility you work in?

- Primary-level hospital

- Secondary-level hospital

- Tertiary-level hospital

What is the approximate size of population served by your hospital?

- < 50,000 inhabitants

- 50,000-199,999 inhabitants

- 200,000-499,999 inhabitants

- 500,000-999,999 inhabitants

- >1,000,000 inhabitants

What is the approximate number of inpatient hospital beds at your facility?

- 0 - 249

- 250 - 499

- 500 - 999

- >=1000

Approximately how many patients with traumatic injuries of any type, involving any part of the body, present to your hospital over an average week? Include patients both admitted to hospital or discharged home

Approximately how many patients with traumatic injuries of any type, involving any part of the body, are admitted to your hospital over an average week? Any patient for who it is decided that they require an inpatient hospital bed for further investigation / management

Approximately how many patients with traumatic injuries of any type, involving any part of the body, undergo an operation at your hospital over an average week? Includes any type of trauma operation (not just abdominal injuries)

- Prehospital care

- In-hospital care

- Rehabilitation

If you had to pick a single phase of trauma management within or outside of your hospital to improve, which do you think could have the greatest impact on patient outcome? Please state the reasons for choosing your given answer

What do you perceive the main limitations to be in accessing care for patients who suffer trauma in your region and require an emergency laparotomy?

Pre-Hospital Phase of Care

Does your hospital ever receive patients with traumatic injuries transferred from another hospital?

- Yes

- No

What is the most common reason for receiving patients from another hospital following traumatic injury?

- For surgical intervention

- For high-level care (e.g. ICU or HDU)

- For rehabilitation

Does your hospital ever transfer out patients with traumatic injuries to another hospital?

- Yes

- No

What is typically the main reason for transferring patients out to hospital following traumatic injury?

- For surgical intervention

- For high-level care (e.g. ICU or HDU)

- For rehabilitation

In your experience, what are the most common reasons for delays in patients arriving to your hospital following their initial injury?

In your experience, if a patient is admitted to hospital requires a trauma laparotomy, what are the most common reasons for delays to getting to theatre?

How often do you have a trauma team who are available to immediately assess seriously injured patients when they first arrive at your hospital?

- All of the time

- Most of the time

- Some of the time

- None of the time

What proportion of trauma patients receiving care in the Emergency Department (or equivalent) would be managed by doctors with formal training in initial trauma care?

- 0% - 24%

- 25% - 49%

- 50% - 74%

- 75% - 100%

Which specialities are present in your hospital for the care and management of trauma patients? At all times; Most of the time; Some of the time; Never

- General Surgery

- Trauma Surgery

- Vascular Surgery

- Orthopaedic Surgery

- Cardiothoracic Surgery

- Plastic Surgery

- Neurosurgery

- Obstetrics & Gynaecology

- Paediatric Surgery

- Anaesthesia

- Intensive Care

In the last 6 months, how often has your hospital had access to a working Computerised Tomography (CT) scan?

- All of the time

- Most of the time

- Some of the time

- None of the time

In the last 6 months, how often has your hospital had access to pathology services?

- All of the time

- Most of the time

- Some of the time

- None of the time

In the last 6 months, how often has your institution been able to provide blood products for transfusion if clinically required?

- All of the time

- Most of the time

- Some of the time

- None of the time

Pre-Operative and Intra-Operative Phase of Care

Approximately how many fully-trained medically-qualified general surgeons are employed by your hospital?

Approximately how many fully-trained medically-qualified specific trauma surgeons are employed by your hospital?

Approximately how many fully-trained medically-qualified anaesthesiologists are employed by your hospital?

Does your hospital ever perform laparoscopic procedures for select trauma patients?

- Yes

- No

(If no, what are the main reasons for not performing laparoscopic procedure on select trauma patients at

your hospital?)

Does your hospital ever perform interventional radiological procedure for select trauma patients?

- Yes

- No

What are the main reasons for not performing interventional radiological procedures on select trauma patients at your institution?

Post-Operative and Rehabilitation Phase of Care

Does your hospital have an intensive care unit? Often termed an ITU or ICU

- Yes

- No

Does your hospital have a high dependency unit? Often termed a HDU

- Yes

- No

In your experience, what are the main barriers that prevent patients being admitted following a trauma laparotomy to an intensive care unit or high dependency unit?

In your experience, for patients that undergo a trauma laparotomy and survive, what is the most common cause for post-operative morbidity?

How often do trauma laparotomy patients have access to a physiotherapist (at least once) after the acute period of their illness?

- All of the time

- Most of the time

- Some of the time

- Never

How often do trauma laparotomy patients have access to an occupational therapist (at least once) after the acute period of their illness?

- All of the time

- Most of the time

- Some of the time

- Never

How often do trauma laparotomy patients have access to a dietician (at least once) after the acute period of their illness?

- All of the time

- Most of the time

- Some of the time

- Never

## Supplementary Material 3 – Characteristics of Included Hospitals

| **Hospital Characteristic** | | **HDI Tertile** | | | **Total**  n=187 |
| --- | --- | --- | --- | --- | --- |
|  |  | **Lower**  n=54 | **Middle**  n=50 | **Higher**  n=83 |  |
| **Location** | Rural | 6 (3.2%) | 4 (2.1%) | 4 (2.1%) | 14 (7.5%) |
|  | Urban | 48 (25.7%) | 46 (24.6%) | 79 (42.2%) | 173 (92.5%) |
| **Facility type** | Public/government | 46 (24.6%) | 46 (24.6%) | 70 (37.4%) | 162 (86.6%) |
|  | Private (for profit) | 3 (1.6%) | 4 (2.1%) | 4 (2.1%) | 11 (5.9%) |
|  | Private (not for profit) | 5 (2.7%) | 0 (0%) | 9 (4.8%) | 14 (7.5%) |
| **Level of facility** | Primary | 2 (1.1%) | 4 (2.1%) | 6 (3.2%) | 12 (6.4%) |
|  | Secondary | 15 (8.0%) | 8 (4.3%) | 13 (7.0%) | 36 (19.3%) |
|  | Tertiary | 37 (19.8%) | 38 (20.3%) | 64 (34.2%) | 139 (74.3%) |
| **Population served** | <50,000 | 2 (1.1%) | 1 (0.5%) | 3 (1.6%) | 6 (3.2%) |
|  | 50,000-199,999 | 7 (3.7%) | 6 (3.2%) | 6 (3.2%) | 19 (10.2%) |
|  | 200,000-499,999 | 6 (3.2%) | 8 (4.3%) | 14 (7.5%) | 28 (15.0%) |
|  | 500,000-999,999 | 12 (6.4%) | 3 (1.6%) | 25 (13.4%) | 40 (21.4%) |
|  | ≥1,000,000 | 27 (14.4%) | 32 (17.1%) | 35 (18.7%) | 94 (50.3%) |
| **Number of inpatient beds** | 0-249 | 17 (9.1%) | 6 (3.2%) | 8 (4.3%) | 31 (16.6%) |
|  | 250-499 | 16 (8.6%) | 12 (6.4%) | 12 (6.4%) | 40 (21.4%) |
|  | 500-999 | 12 (6.4%) | 10 (5.3%) | 39 (20.9%) | 61 (32.6%) |
|  | ≥1,000 | 9 (4.8%) | 22 (11.8%) | 24 (12.8%) | 55 (29.4%) |

## Supplementary Material 4 – Site Survey Quantitative Data

Number of hospitals by rural vs urban location:

| **HDI tertile** | **Rural** | **Urban** |
| --- | --- | --- |
| Lower | 6 | 48 |
| Middle | 4 | 46 |
| Upper | 4 | 79 |

Number of hospitals by funding source:

| **HDI tertile** | **Full government funding** | **Partial government funding** | **Patient insurance** | **Patient Out of Pocket** | **Non-Governmental Organisation** |
| --- | --- | --- | --- | --- | --- |
| Lower | 13 | 23 | 18 | 31 | 10 |
| Middle | 29 | 19 | 20 | 12 | 6 |
| Upper | 65 | 21 | 18 | 13 | 5 |

Number of hospitals by facility type:

| **HDI tertile** | **Public/government** | **Private (for profit)** | **Private (not for profit, e.g. mission hospital)** |
| --- | --- | --- | --- |
| Lower | 46 | 3 | 5 |
| Middle | 46 | 4 | 0 |
| Upper | 70 | 4 | 9 |

Number of hospitals by facility level:

| **HDI tertile** | **Primary** | **Secondary** | **Tertiary** |
| --- | --- | --- | --- |
| Lower | 2 | 15 | 37 |
| Middle | 4 | 8 | 38 |
| Upper | 6 | 13 | 64 |

Number of hospitals by population served (thousands):

| **HDI tertile** | **<50** | **50-199** | **200-499** | **500-999** | **1,000+** |
| --- | --- | --- | --- | --- | --- |
| Lower | 2 | 7 | 6 | 12 | 27 |
| Middle | 1 | 6 | 8 | 3 | 32 |
| Upper | 3 | 6 | 14 | 25 | 35 |

Number of hospitals by number of inpatient beds:

| **HDI tertile** | **0-249** | **250-499** | **500-999** | **1000+** |
| --- | --- | --- | --- | --- |
| Lower | 17 | 16 | 12 | 9 |
| Middle | 6 | 12 | 10 | 22 |
| Upper | 8 | 12 | 39 | 24 |

Number of hospitals by availability of trauma team to assess patients:

| **HDI Tertile** | **All of the time** | **Most of the time** | **Some of the time** | **None of the time** |
| --- | --- | --- | --- | --- |
| Lower | 20 | 22 | 9 | 3 |
| Middle | 30 | 13 | 3 | 4 |
| Upper | 64 | 9 | 8 | 2 |

Number of hospitals by proportion of patients in ED receiving care from a trauma-trained doctor:

| **HDI Tertile** | **0-24%** | **25-49%** | **50-74%** | **75-100%** |
| --- | --- | --- | --- | --- |
| Lower | 9 | 6 | 22 | 17 |
| Middle | 9 | 10 | 11 | 20 |
| Upper | 6 | 8 | 14 | 55 |

Number of hospitals by availability of general surgery:

| **HDI Tertile** | **At all times** | **Most of the time** | **Some of the time** | **Never** |
| --- | --- | --- | --- | --- |
| Lower | 48 | 5 | 1 | 0 |
| Middle | 38 | 8 | 2 | 2 |
| Upper | 78 | 1 | 2 | 2 |

Number of hospitals by availability of orthopaedic surgery:

| **HDI Tertile** | **At all times** | **Most of the time** | **Some of the time** | **Never** |
| --- | --- | --- | --- | --- |
| Lower | 38 | 11 | 4 | 1 |
| Middle | 35 | 11 | 1 | 3 |
| Upper | 72 | 8 | 1 | 2 |

Number of hospitals by availability of obstetrics and gynaecology:

| **HDI Tertile** | **At all times** | **Most of the time** | **Some of the time** | **Never** |
| --- | --- | --- | --- | --- |
| Lower | 41 | 4 | 4 | 5 |
| Middle | 34 | 3 | 9 | 4 |
| Upper | 59 | 7 | 9 | 8 |

Number of hospitals by availability of trauma surgery:

| **HDI Tertile** | **At all times** | **Most of the time** | **Some of the time** | **Never** |
| --- | --- | --- | --- | --- |
| Lower | 20 | 10 | 15 | 9 |
| Middle | 28 | 7 | 8 | 7 |
| Upper | 47 | 13 | 7 | 16 |

Number of hospitals by availability of vascular surgery:

| **HDI Tertile** | **At all times** | **Most of the time** | **Some of the time** | **Never** |
| --- | --- | --- | --- | --- |
| Lower | 11 | 4 | 24 | 15 |
| Middle | 20 | 9 | 13 | 8 |
| Upper | 44 | 19 | 10 | 10 |

Number of hospitals by availability of cardiothoracic surgery:

| **HDI Tertile** | **At all times** | **Most of the time** | **Some of the time** | **Never** |
| --- | --- | --- | --- | --- |
| Lower | 9 | 6 | 13 | 26 |
| Middle | 17 | 4 | 15 | 14 |
| Upper | 36 | 10 | 21 | 16 |

Number of hospitals by availability of plastic surgery:

| **HDI Tertile** | **At all times** | **Most of the time** | **Some of the time** | **Never** |
| --- | --- | --- | --- | --- |
| Lower | 19 | 9 | 13 | 13 |
| Middle | 21 | 4 | 11 | 14 |
| Upper | 34 | 17 | 18 | 14 |

Number of hospitals by availability of neurosurgery:

| **HDI Tertile** | **At all times** | **Most of the time** | **Some of the time** | **Never** |
| --- | --- | --- | --- | --- |
| Lower | 23 | 9 | 12 | 10 |
| Middle | 27 | 11 | 5 | 7 |
| Upper | 52 | 10 | 8 | 13 |

Number of hospitals by availability of paediatric surgeons:

| **HDI Tertile** | **At all times** | **Most of the time** | **Some of the time** | **Never** |
| --- | --- | --- | --- | --- |
| Lower | 27 | 9 | 10 | 8 |
| Middle | 18 | 8 | 11 | 13 |
| Upper | 29 | 11 | 7 | 36 |

Number of hospitals by availability of anaesthetists:

| **HDI Tertile** | **At all times** | **Most of the time** | **Some of the time** | **Never** |
| --- | --- | --- | --- | --- |
| Lower | 42 | 4 | 6 | 2 |
| Middle | 41 | 3 | 5 | 1 |
| Upper | 78 | 4 | 1 | 0 |

Number of hospitals by availability of intensive care medicine:

| **HDI Tertile** | **At all times** | **Most of the time** | **Some of the time** | **Never** |
| --- | --- | --- | --- | --- |
| Lower | 26 | 9 | 13 | 6 |
| Middle | 40 | 7 | 3 | 0 |
| Upper | 75 | 4 | 1 | 3 |

Number of hospitals by access to CT scanning:

| **HDI Tertile** | **At all times** | **Most of the time** | **Some of the time** | **Never** |
| --- | --- | --- | --- | --- |
| Lower | 15 | 26 | 6 | 7 |
| Middle | 30 | 18 | 1 | 1 |
| Upper | 82 | 1 | 0 | 0 |

Number of hospitals by access to pathology:

| **HDI Tertile** | **At all times** | **Most of the time** | **Some of the time** | **Never** |
| --- | --- | --- | --- | --- |
| Lower | 22 | 15 | 12 | 5 |
| Middle | 24 | 13 | 10 | 3 |
| Upper | 76 | 6 | 1 | 0 |

Number of hospitals by access to blood products for transfusion:

| **HDI Tertile** | **At all times** | **Most of the time** | **Some of the time** | **Never** |
| --- | --- | --- | --- | --- |
| Lower | 26 | 23 | 5 | 0 |
| Middle | 25 | 21 | 4 | 0 |
| Upper | 78 | 5 | 0 | 0 |

Number of hospitals by access to laparoscopic procedures for trauma patients:

| **HDI Tertile** | **Yes** | **No** |
| --- | --- | --- |
| Lower | 20 | 34 |
| Middle | 45 | 5 |
| Upper | 80 | 3 |

Number of hospitals by access to interventional radiology:

| **HDI Tertile** | **Yes** | **No** |
| --- | --- | --- |
| Lower | 18 | 36 |
| Middle | 34 | 16 |
| Upper | 73 | 10 |

Number of hospitals by presence of an ICU:

| **HDI Tertile** | **Yes** | **No** |
| --- | --- | --- |
| Lower | 47 | 7 |
| Middle | 49 | 1 |
| Upper | 81 | 2 |

Number of hospitals by presence of HDU:

| **HDI Tertile** | **Yes** | **No** |
| --- | --- | --- |
| Lower | 27 | 27 |
| Middle | 30 | 20 |
| Upper | 60 | 23 |

Number of hospitals by availability of physiotherapy services:

| **HDI Tertile** | **All of the time** | **Most of the time** | **Some of the time** | **Never** |
| --- | --- | --- | --- | --- |
| Lower | 14 | 14 | 19 | 7 |
| Middle | 15 | 17 | 17 | 1 |
| Upper | 41 | 27 | 15 | 0 |

Number of hospitals by availability of occupational therapy services:

| **HDI Tertile** | **All of the time** | **Most of the time** | **Some of the time** | **Never** |
| --- | --- | --- | --- | --- |
| Lower | 2 | 6 | 21 | 25 |
| Middle | 7 | 14 | 17 | 12 |
| Upper | 32 | 12 | 27 | 12 |

Number of hospitals by availability of dietician:

| **HDI Tertile** | **All of the time** | **Most of the time** | **Some of the time** | **Never** |
| --- | --- | --- | --- | --- |
| Lower | 8 | 11 | 19 | 16 |
| Middle | 12 | 15 | 14 | 9 |
| Upper | 37 | 19 | 22 | 5 |


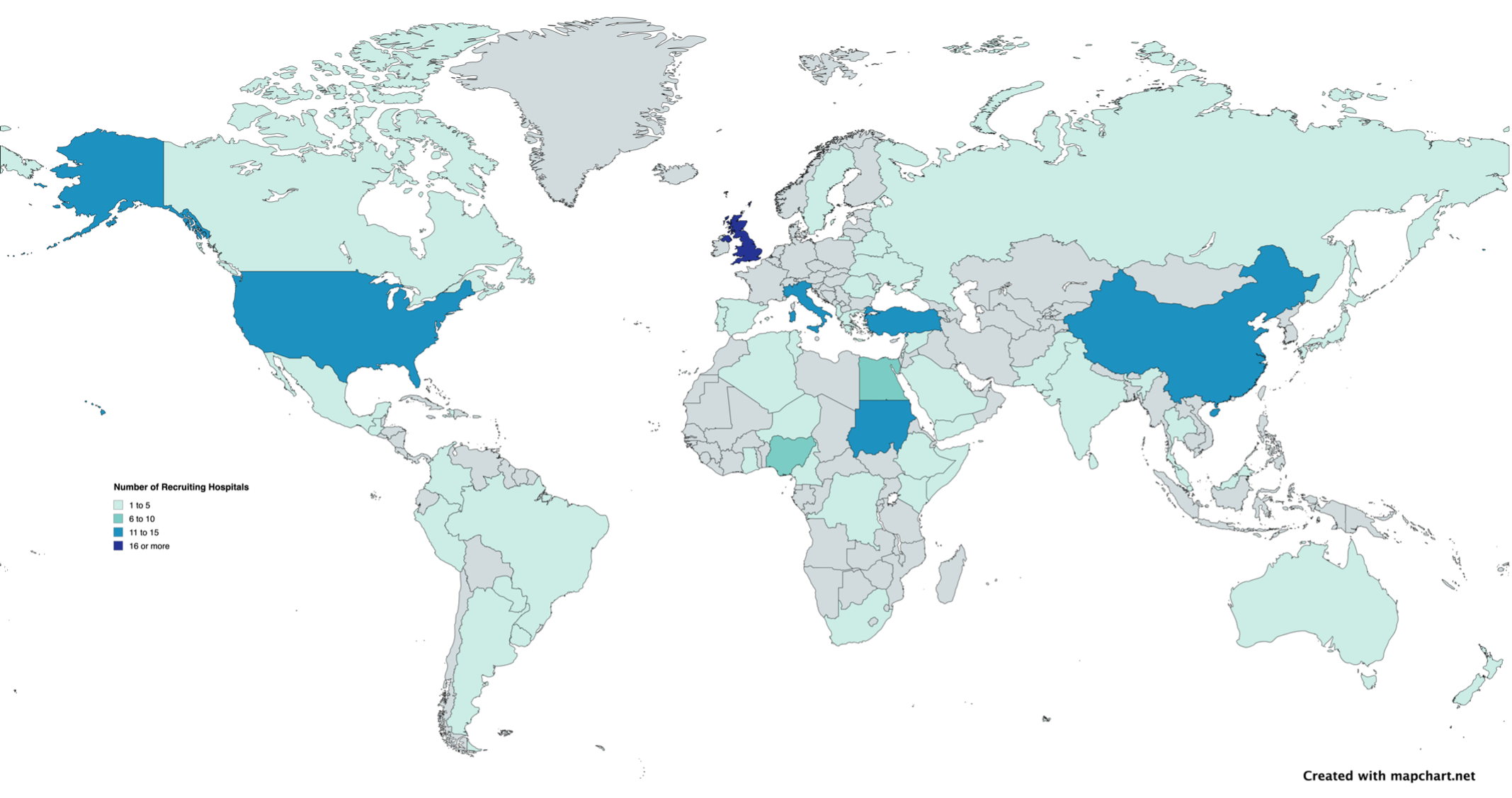


Figure A1 - Global distribution of recruiting centres, demonstrating number of hospitals enrolled per country


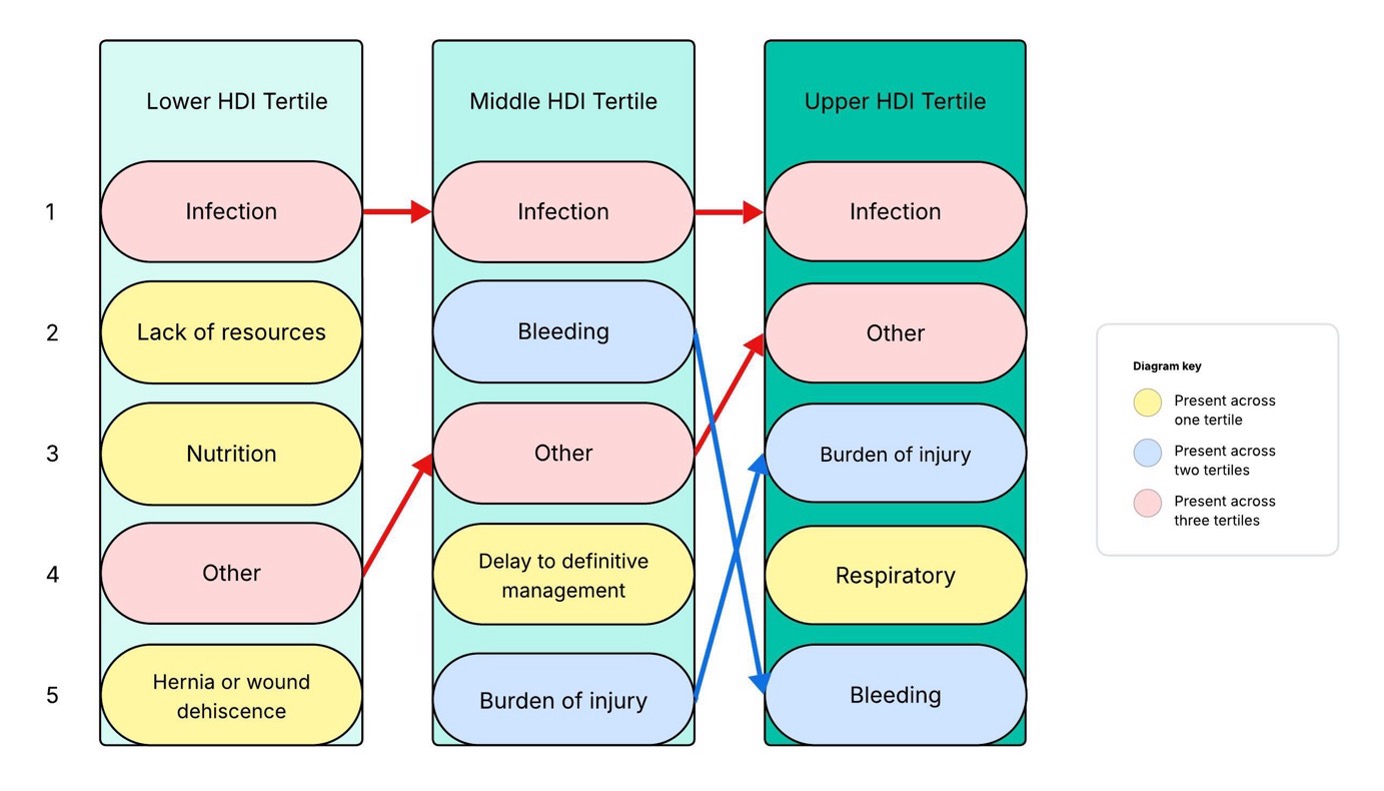


Figure A2 - The most common causes reported by respondents of post-operative morbidity, numbered 1 to 5, stratified by HDI tertile
